# Long-term trends, current status, and transitions of water quality in Chesapeake Bay

**DOI:** 10.1038/s41598-019-43036-6

**Published:** 2019-04-30

**Authors:** Lawrence W. Harding, Michael E. Mallonee, Elgin S. Perry, W. David Miller, Jason E. Adolf, Charles L. Gallegos, Hans W. Paerl

**Affiliations:** 1 Department of Atmospheric and Oceanic Sciences, University of California, Los Angeles, Los Angeles, California 90095 United States; 2 Interstate Commission on the Potomac River Basin, United States Environmental Protection Agency, Chesapeake Bay Program Office, 410 Severn Avenue, Annapolis, Maryland 21403 United States; 3 Statistics Consultant, 377 Resolutions Rd., Colonial Beach, Virginia, 22443 United States; 4 U.S. Naval Research Laboratory, 4555 Overlook Ave., SW, Washington, DC 20375 United States; 5 Department of Biology, Monmouth University, West Long Branch, NJ 07764 United States; 6 Smithsonian Environmental Research Center, 647 Contees Wharf Road, Edgewater, Maryland 21037 United States; 7 Institute of Marine Sciences, University of North Carolina at Chapel Hill, 3431 Arendell Street, Morehead City, North Carolina 28557 United States

**Keywords:** Ecology, Ocean sciences

## Abstract

Coincident climatic and human effects strongly influence water-quality properties in estuarine-coastal ecosystems around the world. Time-series data for a number of ecosystems reveal high spatio-temporal variability superimposed on secular trends traceable to nutrient over-enrichment. In this paper, we present new analyses of long-term data for Chesapeake Bay directed at several goals: (1) to distinguish trends from spatio-temporal variability imposed by climatic effects; (2) to assess long-term trends of water-quality properties reflecting degradation and recovery; (3) to propose numerical water-quality criteria as targets for restoration; (4) to assess progress toward attainment of these targets. The bay has experienced multiple impairments associated with nutrient over-enrichment since World War II, e.g., low dissolved oxygen (DO), decreased water clarity, and harmful algal blooms (HAB). Anthropogenic eutrophication has been expressed as increased chlorophyll*-a* (*chl-a*) driven by accelerated nutrient loading from 1945 to 1980. Management intervention led to decreased loading thereafter, but deleterious symptoms of excess nutrients persist. Climatic effects exemplified by irregular “dry” and “wet” periods in the last 30+ years largely explain high inter-annual variability of water-quality properties, requiring adjustments to resolve long-term trends. Here, we extend these analyses at a finer temporal scale to six decades of *chl-a*, Secchi depth, and nitrite plus nitrate (NO_2_ + NO_3_) data to support trend analyses and the development of numerical water-quality criteria. The proposed criteria build on a conceptual model emphasizing the need to distinguish climatic and human effects in gauging progress to reverse eutrophication in estuarine-coastal ecosystems.

## Introduction

Significant changes in estuarine-coastal ecosystems around the world can be traced to climatic and anthropogenic effects^1–4^. These changes are manifested as secular trends of water-quality properties driven by human behavior against a backdrop of spatio-temporal variability associated primarily with regional climate fluctuations^5^. Long-term data for such ecosystems were limited prior to the 1960s^6^ compared to more extensive records for marine and terrestrial ecosystems that supported development of basic ecological concepts^7^. Recent analyses of multi-decadal time series have proven effective to identify secular changes for a diverse set of estuarine-coastal ecosystems, including Narragansett Bay in Rhode Island (USA)^8^, Chesapeake Bay in the mid-Atlantic (USA)^9^, the Neuse River estuary in North Carolina (USA)^10^, Tampa Bay in Florida (USA)^11^, the San Francisco Bay estuary in California (USA)^12^, the Baltic Sea in northern Europe^13^, and the northern Adriatic Sea in southern Europe^14^. These studies and others supported a global synthesis for ecosystems at the land-sea margin, focusing on long-term trends and major drivers of spatio-temporal variability^5^.

Progress to define changes in estuarine-coastal ecosystems has benefited from systematic monitoring of water-quality properties, stimulated by efforts to reverse environmental degradation. Several properties are instrumental for assessing estuarine health, including: (1) chlorophyll-*a* (*chl-a*) as a measure of phytoplankton biomass; (2) Secchi depth as a proxy for water clarity; (3) nitrite plus nitrate (NO_2_ + NO_3_) as a measure of nutrient loading/concentrations. Current thinking based on analyses of data aggregated at an annual time scale includes widespread recognition of climatic and human effects as drivers of change, with perturbations by tropical storms, drought-flood cycles, and irregular “dry” and “wet” periods superimposed on long-term trends of water-quality properties^5,6^.

Our group has focused on Chesapeake Bay, a large, temperate estuary in the mid-Atlantic region of the United States where nutrient over-enrichment has produced multiple symptoms of anthropogenic eutrophication^3^. An upward “trajectory” of eutrophication since World War II is evident in time series of total nitrogen (TN) and nitrite plus nitrate (NO_2_ + NO_3_) loading^15,16^, recently analyzed using flow-adjusted loading to account for climatic effects on hydrology^17,18^. These analyses showed a doubling of TN and NO_2_ + NO_3_ loading from 1945 to the early 1980s, with TN loading increasing >120% and NO_2_ + NO_3_ loading increasing 90%. Modest progress to reverse anthropogenic eutrophication consists of decreased TN loading of ~19% and NO_2_ + NO_3_ loading of ~5% from 1981 to 2012. While reduced TN and NO_2_ + NO_3_ loading in the last 30+ years suggests the beginning of recovery despite a 34% increase of the human population since 1985 (13.5 to 18.1 million), improvements of water quality and a reversal of detrimental biotic effects have yet to occur.

**Figure 1:**
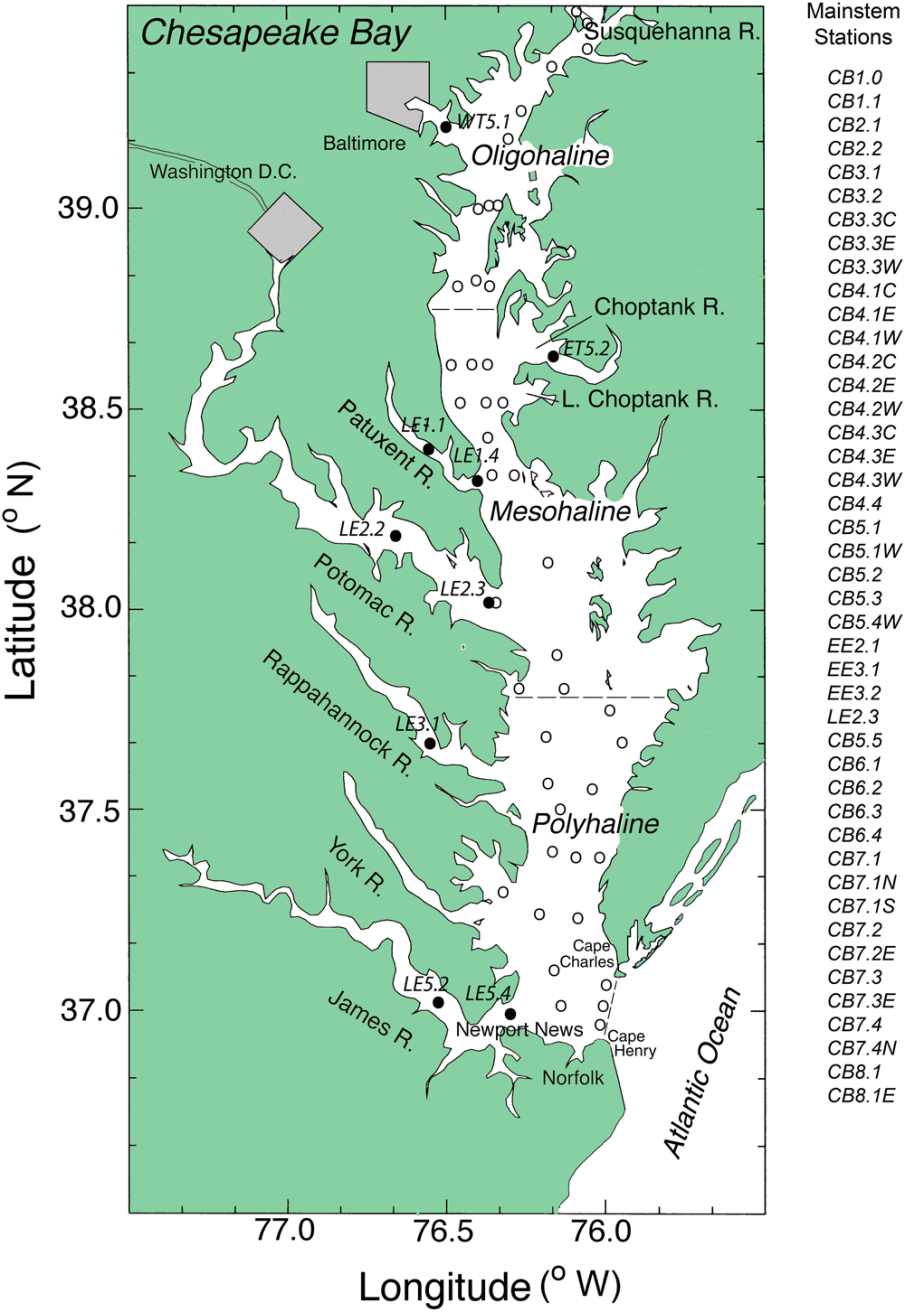
Chesapeake Bay showing major rivers, cities, salinity zones, and sampling stations for water-quality observations. Main-stem bay stations are indicated by open circles; tributary stations are indicated by closed circles. The study-site map was generated with the software package Surfer (Golden Software) and modified using Photoshop (Adobe).

Previous analyses in Chesapeake Bay largely focused on annual-scale properties and processes to resolve long-term trends from spatio-temporal variability^17,18^, following the approach in global syntheses for estuarine-coastal ecosystems^5^. We used freshwater discharge and a synoptic climatology for the mid-Atlantic region of the United States (USA) to quantify hydrological forcing^19–21^, allowing us to adjust for climatic effects. The bay is a large, intricate ecosystem with highly variable freshwater inflow dominated by the Susquehanna River, supplemented by lateral inputs from a number of tributaries, including in order of annual discharge the Potomac, James, Rappahannock, Patuxent, Choptank, and Patapsco Rivers (Fig. 1). Annual cycles of phytoplankton biomass and production are primarily regulated by light- and nitrogen (N) limitation on the scale of the bay^22^, contrasted with seasonal phosphorus (P) limitation in tidal-fresh and oligohaline (OH) salinity zones^23^. Consistent with the common use of annual means to define overall patterns and trends^5^, we documented climatic and human effects on phytoplankton dynamics in the bay using mean, annual *chl-a*, net primary productivity (NPP), cell-size distribution, and floral composition^17,18,24^.

Despite significant progress in comparative studies of estuarine-coastal ecosystems using annual means, analyses at finer temporal resolution promise additional insights on seasonal variability. In previous studies, we used multiple lines of scientific evidence to develop numerical *chl-a* criteria for the bay^25^. Here, we extend those analyses to propose numerical criteria for *chl-a*, Secchi depth, and NO_2_ + NO_3_ based on data aggregated at monthly to seasonal scales, with applications to assess long-term trends, current status, and transitions of water quality. Source data for freshwater flow, TN and NO_3_ loading, *chl-a*, Secchi depth, and NO_2_ + NO_3_ concentrations from the 1960s to 2015 supported these analyses. The statistical approach consisted of generalized additive models (GAM) that allowed us to adjust for climatic effects on water-quality properties, generating time series of flow-adjusted model predictions^17,18^. This approach was directed at several goals: (1) to distinguish trends from spatio-temporal variability imposed by climatic effects; (2) to assess long-term trends of water-quality properties reflecting degradation and recovery; (3) to propose numerical water-quality criteria as targets for restoration; (4) to assess progress at attaining targets.

## Results

### Freshwater flow/climate

**Figure 2:**
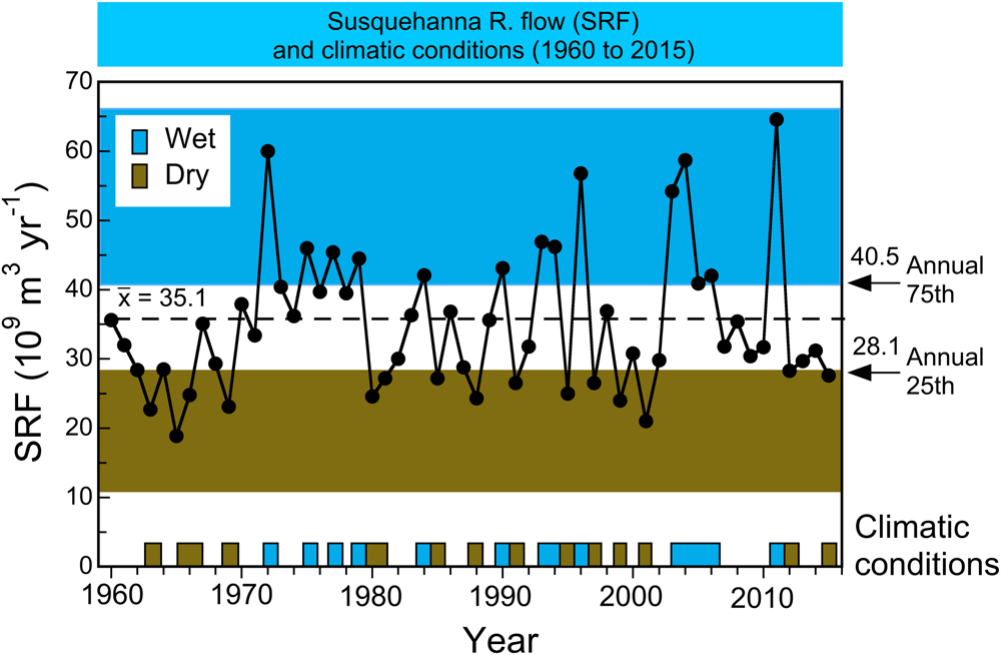
Annual freshwater flow from the Susquehanna River for years corresponding to water-quality observations analyzed here from 1964 to 2015, indicating “dry” and “wet” periods based on 25^th^ and 75^th^ percentiles. Data were obtained from the U.S. Geological Survey. This figure is similar to Figure 2 in Harding *et al*.^18^, modified to cover the time frame for data analyzed in this paper.

The Susquehanna River entering the head of the estuary is the largest source of freshwater to Chesapeake Bay (Fig. 1). Historical records for Susquehanna River flow (SRF) showed high inter-annual variability from 1960 to 2015, the period corresponding to observations of water-quality properties analyzed here (Fig. 2). Hydrological records of annual SRF are presented as the mean, 25^th^, and 75^th^ percentiles to document this variability, identifying “dry” and “wet” periods, including successive decades of drought (1960s) and flood (1970s) conditions. Discharges of other bay tributaries were correlated with that of the Susquehanna River, making annual SRF a useful surrogate for regional climatic effects on the bay’s watershed.

### Model fits of water-quality properties

**Figure 3:**
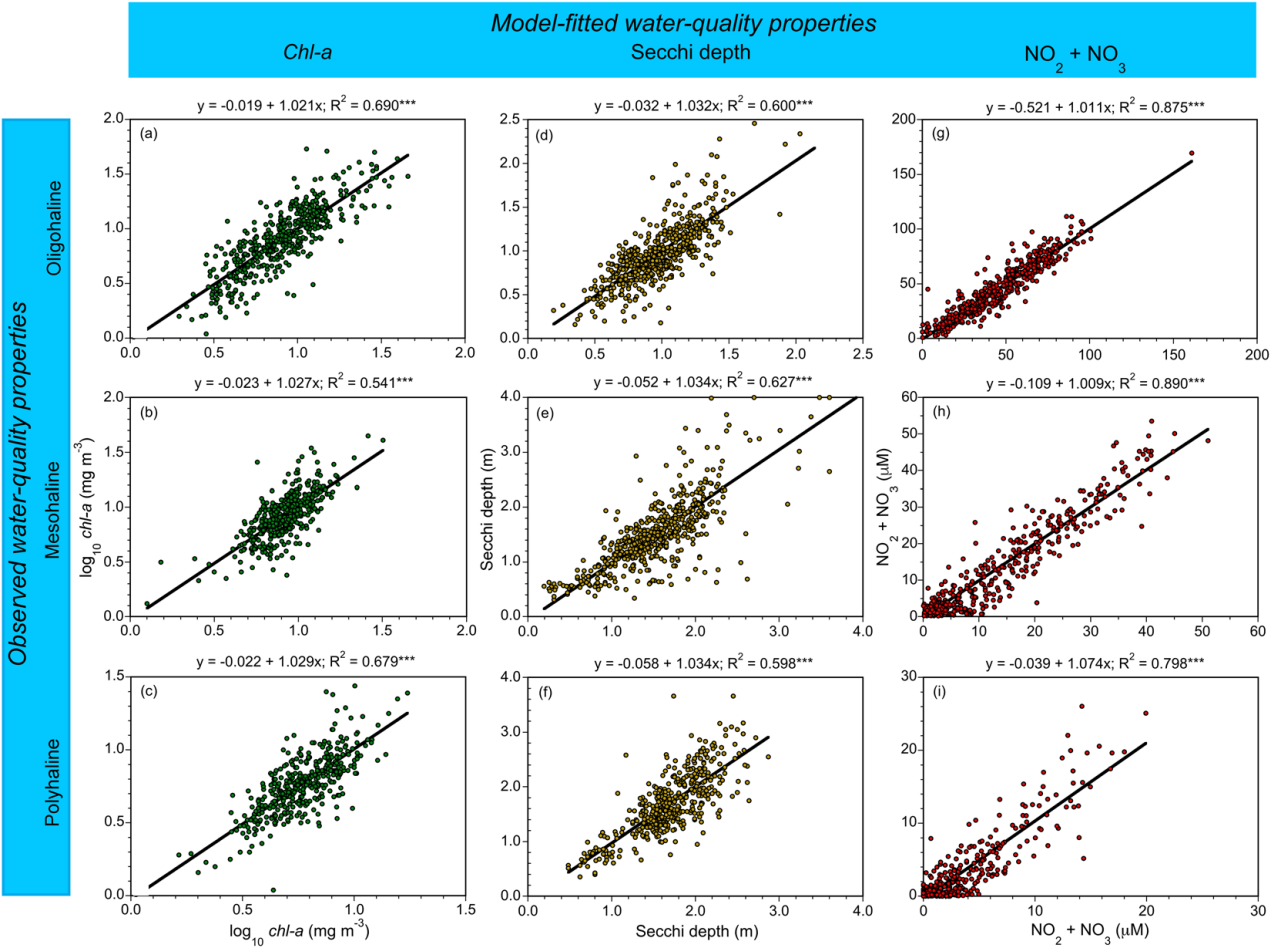
(**a**–**i**) Observed vs model fits of mean, monthly log_10_ *chl-a* (mg m^−3^), Secchi depth (m), and NO_2_ + NO_3_ (μM) for OH, MH, and PH salinity zones in the main-stem bay from 1964 to 2015.

Source data were obtained for stations representing a broad range of conditions in the main-stem bay and its tributaries. Long-term means of salinity, *chl-a*, Secchi depth, and NO_2_ + NO_3_ summarize water-quality properties for these stations (Table 1). Statistical models on monthly, seasonal, and annual scales were developed using GAM, resulting in predictions of log_10_ *chl-a*, Secchi depth, and NO_2_ + NO_3_. Table 2 lists predictor variables of water-quality properties, and Table 3 depicts corresponding model statistics. Our selection of water-quality properties was based on their common use as indicators of anthropogenic eutrophication in estuarine-coastal ecosystems, and we recognize these variables are not independent of one another. Model fits as simple, linear regressions of observed vs predicted log_10_ *chl-a*, Secchi depth, and NO_2_ + NO_3_ for oligohaline (OH), mesohaline (MH), and polyhaline (PH) salinity zones were significant (p < 0.001) and exhibited negligible bias, confirmed by near-unity regression slopes (Fig. 3a–i). Analogous regressions of observed water-quality properties on model-fitted values for nine tributary stations are presented in Supplementary Material.

**Table 1:** Long-term means of water-quality properties from 1985 to 2015 at main-stem bay and tributary stations used in these analyses.

Station	Total depth	Salinity	*chl-a*	Secchi depth	NO_2_ + NO_3_	Station	Total depth	Salinity	*chl-a*	Secchi depth	NO_2_ + NO_3_
**Oligohaline**						**Polyhaline**					
CB1.0	6.0	0.00	5.52	ND	78.0	CB5.4W	5.1	15.2	9.74	1.53	4.83
CB1.1	6.0	0.00	8.03	0.91	80.7	CB5.5	18.2	16.2	9.27	1.92	5.77
CB2.1	6.3	0.48	8.35	0.64	67.5	CB6.1	12.8	16.9	9.69	1.78	4.75
CB2.2	12.3	2.21	6.32	0.68	60.8	CB6.2	10.8	17.6	9.15	1.69	3.99
CB3.1	13.2	4.53	9.76	0.74	51.3	CB6.3	11.9	18.1	9.19	1.52	3.41
CB3.2	12.1	6.72	12.1	0.90	41.7	CB6.4	10.0	19.9	8.44	1.67	2.32
CB3.3C	24.1	9.31	15.7	1.08	30.0	CB7.1	23.0	18.2	8.99	1.69	3.06
CB3.3E	8.2	9.26	17.3	1.03	25.3	CB7.1N	29.3	17.1	10.0	1.53	3.95
CB3.3W	9.0	9.05	19.2	0.87	25.7	CB7.1S	15.7	18.2	8.43	1.84	3.54
CB4.1C	32.3	11.2	12.6	1.37	23.0	CB7.2	21.7	18.9	7.96	1.85	3.08
CB4.1E	23.8	11.4	12.3	1.35	19.4	CB7.2E	13.2	20.3	7.55	1.79	2.17
CB4.1W	9.3	10.6	19.9	1.06	21.4	CB7.3	13.6	22.0	6.19	1.94	1.71
**Mesohaline**						CB7.3E	18.6	22.5	6.86	1.86	1.45
						CB7.4	14.2	25.3	5.33	2.15	1.14
						CB7.4N	12.3	27.4	4.65	2.04	0.80
CB4.2C	27.2	12.1	10.3	1.60	18.3	CB8.1	9.6	21.7	8.01	1.55	2.03
CB4.2E	9.4	12.0	10.7	1.60	16.2	CB8.1E	16.6	23.7	6.70	1.70	1.54
CB4.2W	9.4	11.6	17.4	1.29	17.2	**Tributaries**					
CB4.3C	26.9	12.4	9.52	1.68	17.5						
CB4.3E	22.3	12.4	9.75	1.61	15.4						
CB4.3W	9.8	11.9	15.4	1.37	16.2	WT5.1	15.4	7.65	34.4	0.84	36.5
CB4.4	30.6	13.1	11.2	1.56	14.4	ET5.2	11.6	9.74	13.1	0.98	20.8
CB5.1	31.6	13.7	9.94	1.68	13.4	LE1.1	11.9	10.9	18.2	0.99	5.8
CB5.1W	9.3	13.4	10.6	1.56	13.7	LE1.4	15.6	12.8	13.0	1.53	10.9
CB5.2	30.6	14.2	9.59	1.76	10.6	LE2.2	12.6	10.7	15.5	1.32	15.4
CB5.3	26.8	14.5	9.75	1.72	9.25	LE2.3	20.1	13.1	10.4	1.71	11.4
CB5.4	32.5	15.4	9.82	1.91	7.10	LE3.1	6.6	12.5	13.0	1.02	5.2
						LE5.2	8.8	10.6	11.4	0.90	13.5
						LE5.4	15.9	19.1	9.04	1.19	5.9

**Table 2:** Predictor variables for generalized additive models (GAM) of water-quality properties − log_10_ *chl-a*, Secchi depth, and NO_2_ + NO_3 _− in Chesapeake Bay.

Predictor variables
log_10_ SRF (mean, monthly)
log_10_ SUM (cumulative, monthly)
salinity (mean, monthly within a salinity zone)
sequential month (from start of time series)
month (within a year)
season
year
decade
TN loading (annual)
NO_2_ + NO_3_ loading (annual)

**Table 3:** Statistics for generalized additive models (GAM) of water-quality properties in Chesapeake Bay using the set of predictor variables compiled in Table 1.

Water-quality property	Time frame	R^2^ (adjusted)	% Deviance explained	GCV^a^
**log**_**10**_ ***chl-a***				
OH	1964–2015	0.654	69.0	0.0349
MH	1964–2015	0.492	55.9	0.0273
PH	1965–2015	0.614	67.9	0.0236
**Secchi depth**				
OH	1964–2015	0.553	59.6	0.0604
MH	1960–2015	0.585	62.6	0.2501
PH	1965–2015	0.545	59.8	0.1625
**NO**_**2**_ + **NO**_**3**_				
OH	1964–2015	0.851	87.4	108
MH	1964–2015	0.865	89.0	27.9
PH	1965–2015	0.734	78.2	6.97

^a^GCV = generalized cross-validation score^57–59^.

### Modeling climatic effects

**Figure 4:**
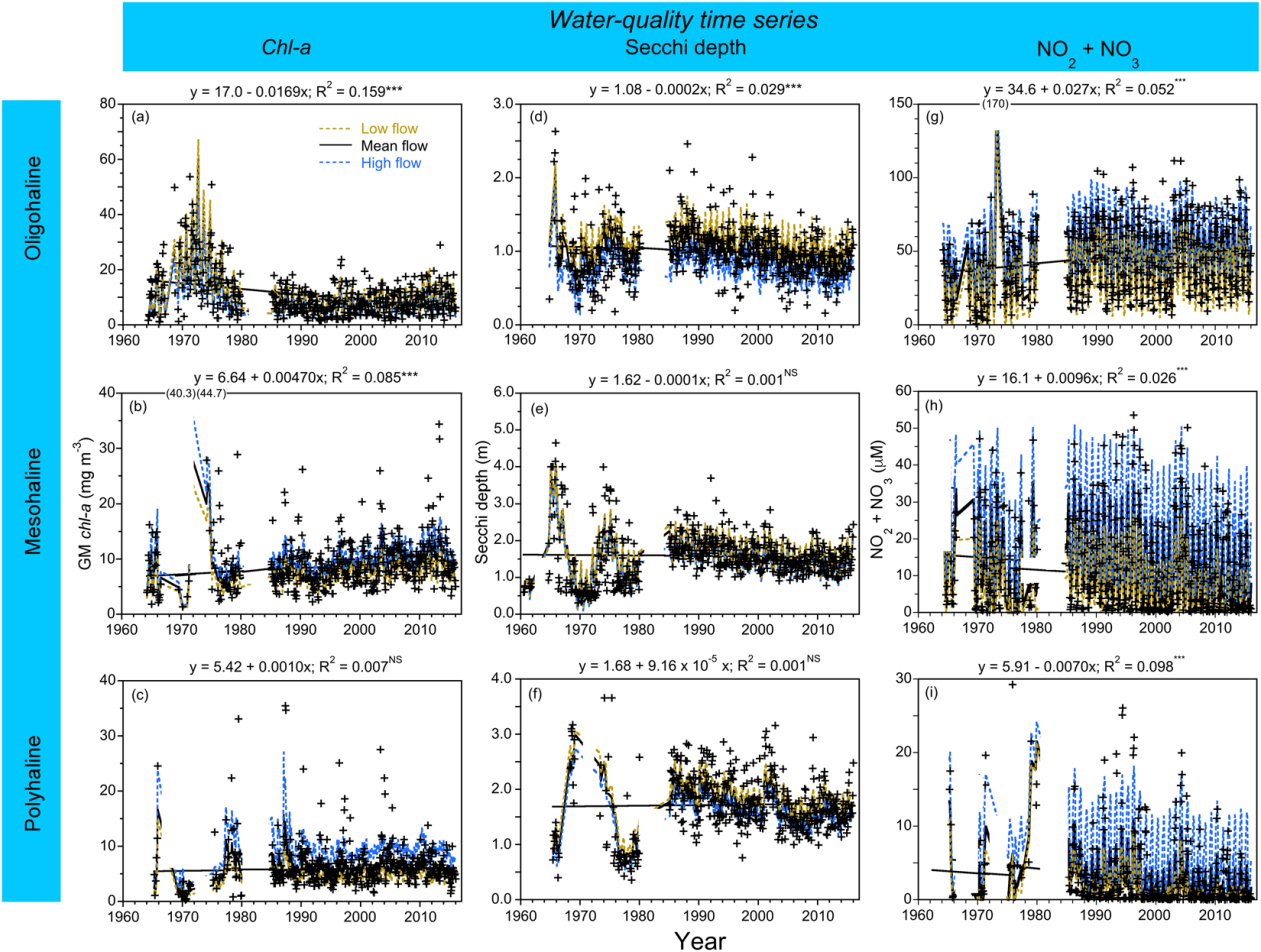
(**a**–**i**) Time-series data from 1964 to 2015 of flow-adjusted model predictions of mean, monthly *chl-a* (geometric mean), Secchi depth, and NO_2_ + NO_3_ for OH, MH, and PH salinity zones in the main-stem bay. *Crosses* show observed values; *solid black lines* depict model predictions in mean-flow conditions; *dashed blue lines* show predictions in high-flow conditions; *dashed brown lines* show predictions in low-flow conditions.

Model predictions of log_10_ *chl-a*, Secchi depth, and NO_2_ + NO_3_ in low-flow, mean-flow, and high-flow conditions were developed as time series by applying GAM to long-term data to adjust for climatic effects (Fig. 4a–i). These climatic effects were computed by varying input terms for SRF and salinity for each salinity zone. Model predictions in mean-flow conditions were obtained by setting flow and salinity terms at their respective means, “dry” conditions with flow and salinity terms set at 10^th^ and 90^th^ percentiles, and “wet” conditions with flow and salinity terms set at 90^th^ and 10^th^ percentiles. Model inputs also included terms for annual TN and NO_2_ + NO_3_ loading to account for inter-annual variability of N-limitation^17,18^. Temporal lags were tested using auto-regression terms (AR) in generalized additive mixed models (GAMM), revealing no significant differences from GAM. Additional details on models are provided in Methods.

Model predictions of mean, monthly *chl-a* for the OH salinity zone in mean-flow conditions showed higher *chl-a* in the 1960s and 1970s than in recent years, with lower *chl-a* after the mid- to late-1970s that continued from 1985 to 2015 (Fig. 4a). Climatic effects on *chl-a* for the OH salinity zone were expressed as model predictions in low-flow, “dry” conditions that exceeded those in mean-flow or high-flow, “wet” conditions. This stimulatory effect on *chl-a* for the OH salinity zone reflected an alleviation of light-limitation as inputs of bio-optically active materials (e.g., suspended particulate matter, chromophoric dissolved organic material) were reduced in low-flow, “dry” conditions. Model predictions of mean, monthly *chl-a* for the OH salinity zone in mean-flow conditions showed a significant downward trend after the mid-1960s. Previous analyses based on long-term data aggregated at an annual scale also showed this step-down of *chl-a* for the OH salinity zone, ascribed to regulation of orthophosphate (PO_4_^3−^), increased P-limitation in the upper bay, and reduction of N uptake in the OH salinity zone leading to increased throughput of TN and NO_2_ + NO_3_ to MH and PH salinity zones^17,18^.

Model predictions of mean, monthly *chl-a* for the MH salinity zone in mean-flow conditions were highly variable, characterized by a shallow, increasing trend that continued throughout the time series (Fig. 4b). Flow-adjusted model predictions for the MH salinity zone showed higher *chl-a* in high-flow, “wet” conditions than in mean-flow or low-flow, “dry” conditions. This climatic effect for the MH salinity zone was opposite that for the OH salinity zone, a pattern consistent with increased TN and NO_2_ + NO_3_ loading in “wet” years, an alleviation of N-limitation, and stimulation of *chl-a* and NPP described previously^17,18^.

Fewer observations were available to support model predictions of mean, monthly *chl-a* for the PH salinity zone than for OH and MH salinity zones, although temporal variability of *chl-a* was comparable among the three salinity zones (Fig. 4c). Climatic effects consisted of higher *chl-a* in high-flow, “wet” conditions than in mean-flow or low-flow, “dry” conditions, similar to the MH salinity zone. Model predictions of mean, monthly *chl-a* for the PH salinity zone in mean-flow conditions did not indicate a secular trend for the period of record (Fig. 4c).

Climatic effects on Secchi depth were expressed as higher model predictions (=increased water clarity) in low-flow, “dry” conditions than in mean-flow or high-flow, “wet” conditions for OH, MH, and PH salinity zones (Fig. 4d–f). Model predictions of Secchi depth in mean-flow conditions showed decreasing trends (=decreased water clarity) for OH and MH salinity zones (Fig. 4d–e), but no trend for the PH salinity zone (Fig. 4f). Model predictions of Secchi depth in high-flow, “wet” conditions were generally lower (=decreased water clarity) than predictions in mean-flow or low-flow, “dry” conditions, but overall, Secchi depth was less sensitive to climatic effects than *chl-a*.

Model predictions of NO_2_ + NO_3_ showed strong climatic effects for OH, MH, and PH salinity zones (Fig. 4g–i). NO_2_ + NO_3_ was consistently higher in high-flow, “wet” conditions than in mean-flow or low-flow, “dry” conditions for all salinity zones. Model predictions of NO_2_ + NO_3_ in mean-flow conditions showed mixed trends, consisting of a shallow increase for the OH salinity zone, and decreases for MH and PH salinity zones. These trends were consistent with decreased N loading since the early 1980s^17,18^.

**Figure 5:**
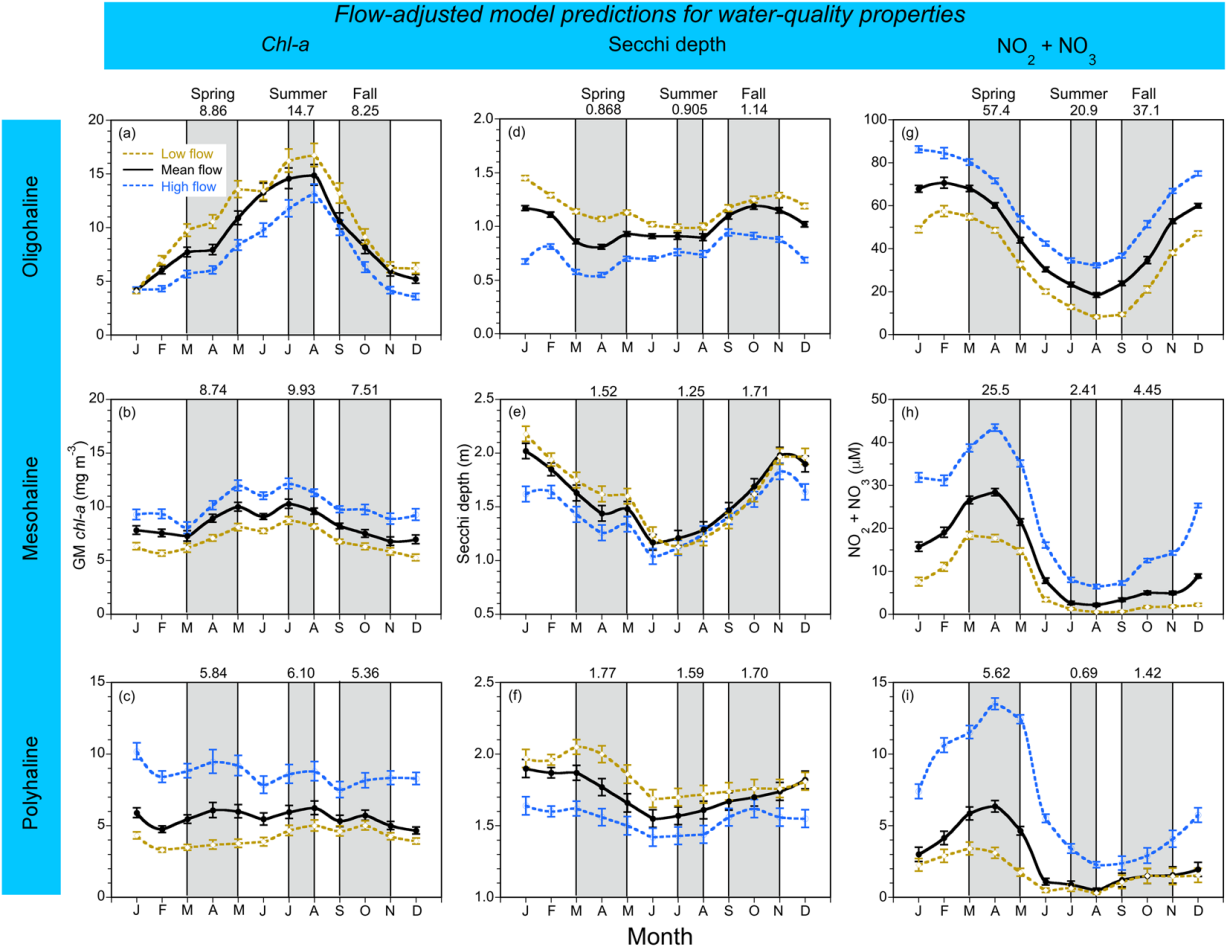
(**a**–**i**) Mean, monthly flow-adjusted model predictions of *chl-a* (geometric mean), Secchi depth, and NO_2_ + NO_3_ aggregated from long-term data from 1964 to 2015 for OH, MH, and PH salinity zones in the main-stem bay. *Solid black lines* depict model predictions in mean-flow conditions; *dashed blue lines* show predictions in high-flow conditions; *dashed brown lines* show predictions in low-flow conditions. Error bars are ±1 standard error (SE) of the estimates. Vertical shaded areas denote spring, summer and fall, with seasonal means depicted on each panel.

Climatic effects on *chl-a*, Secchi depth, and NO_2_ + NO_3_ derived as model predictions in low-flow, “dry” conditions, mean-flow conditions, and high-flow, “wet” conditions were aggregated at a monthly scale (Fig. 5a–i), revealing patterns consistent with predictions for the complete time series (Fig. 4a–i). Mean, monthly *chl-a* for the OH salinity zone was higher in low-flow, “dry” conditions than in mean-flow or high-flow, “wet” conditions (Fig. 5a), and higher for MH and PH salinity zones in high-flow, “wet” conditions (Fig. 5b,c). Climatic effects on other water-quality properties consisted of higher Secchi depth in low-flow, “dry” conditions (Fig. 5d–f), and higher NO_2_ + NO_3_ in high-flow, “wet” conditions for OH, MH, and PH salinity zones (Fig. 5g–i).

### Numerical water-quality criteria

Model predictions aggregated at a monthly scale were used to develop numerical water-quality criteria adjusted for climatic effects. We averaged predictions for spring (March–May), summer (July–Aug), and fall (Sep–Nov) to obtain seasonal values, with predictions in mean-flow conditions shown at the top of each panel (Fig. 5a–i). Numerical criteria for *chl-a*, Secchi depth, and NO_2_ + NO_3_ based on predictions for specific time periods were tabulated as entries for each water-quality property (Tables 4–6). These entries provide the temporal basis of each criterion, consisting of: (1) model predictions in mean-flow conditions for all data (1960s to 2015); (2) model predictions in mean-flow conditions for the 1960s; (3) previously published numerical criteria for *chl-a*^25^; (4) specified model predictions for previous (1985 to 1989) and recent years (2011 to 2015); (5) and percent changes required to attain each criterion.

**Table 4:** Numerical criteria based on flow-adjusted model predictions of *chl-a* (units - mg m^−3^) in Chesapeake Bay aggregated at a monthly scale to account for climatic effects on water-quality properties.

Water-quality property	Salinity zone	Season	Criteria			Recent 2011–2015^d^	Percent differences^e^		
			All data^a^	1960s^b^	Ref.^25^ ^c^				
*chl-a*	OH	Spring	8.86	**5**.**68**	5.90	10.6	19.6	86.6	79.7
	OH	Summer	14.7	**13**.**9**	15.0	12.8	(−12.9)	(−7.91)^f^	(−14.7)
	OH	Fall	8.25	**10**.**7**	ND	7.84	(−4.97)	(−26.7)	ND
	MH	Spring	8.74	**4**.**68**	2.60	11.6	32.7	148	346
	MH	Summer	9.93	**8**.**69**	7.20	11.9	19.8	36.9	65.3
	MH	Fall	7.51	**10**.**3**	ND	8.31	10.7	(−19.3)	ND
	PH	Spring	5.84	**2**.**21**	1.40	5.58	(−4.45)	152	299
	PH	Summer	6.10	**4**.**31**	1.60	6.19	1.48	43.6	287
	PH	Fall	5.36	**6**.**42**	ND	5.59	4.29	(−12.9)	ND

^a^Flow-adjusted model predictions for all data from 1960s to 2015 in mean-flow conditions;

^b^Data extracted from flow-adjusted model predictions for the 1960s in mean-flow conditions;

^c^Published *chl-a* criteria for 1960s in mean-flow predictions from Harding *et al*.^25^;

^d^Data extracted from flow-adjusted model predictions in recent years (2011 to 2015) in mean-flow conditions;

^e^Decreases as percent differences required to attain *chl-a* in recent years (2011 to 2015) in mean-flow conditions; percent differences = ((Recent − Criterion)/Criterion) * 100.

^f^Negative percent differences indicate recent values met specified numerical criteria.

**Table 5:** Numerical criteria based on flow-adjusted model predictions of Secchi depth (units – m) in Chesapeake Bay aggregated at a monthly scale to account for climatic effects on water-quality properties.

Water-quality property	Salinity zone	Season	Criteria			Recent 2011–2015^d^	Percent differences^e^		
			All data^a^	1960s^b^	1985–89^c^				
*Secchi depth*	OH	Spring	0.868	**0**.**680**	1.03	0.724	16.6	(−6.47)^f^	29.7
	OH	Summer	0.905	**0**.**946**	1.06	0.753	20.7	20.4	29.0
	OH	Fall	1.14	**1**.**17**	1.32	1.02	10.5	12.8	22.7
	MH	Spring	1.51	**1**.**97**	1.82	1.37	9.27	30.5	24.7
	MH	Summer	1.25	**1**.**62**	1.56	1.15	8.00	29.0	26.3
	MH	Fall	1.71	**1**.**95**	2.00	1.62	5.25	16.9	19.0
	PH	Spring	1.77	**1**.**70**	1.96	1.70	3.95	0	13.3
	PH	Summer	1.59	**1**.**72**	1.91	1.53	3.77	11.1	19.9
	PH	Fall	1.70	**1**.**90**	2.09	1.63	4.12	14.2	22.0

^a^Flow-adjusted model predictions for all data from the 1960s to 2015 in mean-flow conditions;

^b^Data extracted from flow-adjusted model predictions for the 1960s in mean-flow conditions;

^c^Data extracted from flow-adjusted model predictions for 1985 to 1989 in mean-flow conditions;

^d^Data extracted from flow-adjusted model predictions in recent years (2011 to 2015) in mean-flow conditions;

^e^Decreases as percent differences required to attain *Secchi depth* in recent years (2011 to 2015) in mean-flow conditions; percent differences = (Recent−Criterion/Criterion) * 100; negative values indicate criterion has been attained as increased *Secchi depth* coincides with increased water clarity.

^f^Negative percent differences indicate recent values met specified numerical criteria.

**Table 6:** Numerical criteria based on flow-adjusted model predictions of NO_2_ + NO_3_ (units – μM) in Chesapeake Bay aggregated at a monthly scale to account for climatic effects on water-quality properties.

Water-quality property	Salinity zone	Season	Criteria			Recent	Percent differences^e^		
			All data^a^	1960s^b^	2011–2015^c^	2011–2015^d^			
*NO*_*2*_ + *NO*_*3*_	OH	Spring	57.4	39.4	**48**.**0**	60.0	4.53	52.3	25.0
	OH	Summer	20.9	10.9	**11**.**9**	24.0	14.8	120	102
	OH	Fall	37.1	23.6	**22**.**7**	39.8	7.28	68.6	75.3
	MH	Spring	25.5	29.3	**10**.**8**	19.4	(−23.9)^f^	(−33.8)	79.6
	MH	Summer	2.41	3.88	**ND**	0.733	(−70.0)	(−81.1)	ND
	MH	Fall	4.45	6.47	**0**.**225**	2.39	(−46.3)	(−63.1)	962
	PH	Spring	5.62	7.03	**0**.**780**	3.50	(−37.7)	(−50.2)	349
	PH	Summer	0.690	0.270	**ND**	ND	ND	ND	ND
	PH	Fall	1.42	0.869	**0**.**388**	0.442	(−68.9)	(−49.1)	13.9

^a^Flow-adjusted model predictions for all data from the 1960s to 2015 in mean-flow conditions;

^b^Data extracted from flow-adjusted model predictions for the 1960s in mean-flow conditions;

^c^Data extracted from flow-adjusted model predictions in recent years (2011 to 2015) in low-flow conditions;

^d^Data extracted from flow-adjusted model predictions in recent years (2011 to 2015) in mean-flow conditions;

^e^Decreases as percent differences required to attain *NO*_*2*_ + *NO*_*3*_ in recent years (2011 to 2015) in mean-flow conditions; percent differences = (Recent − Criterion/Criterion) * 100.

^f^Negative percent differences indicate recent values met specified numerical criteria.

Long-term trends and current status of water-quality properties were assessed by comparing model predictions in mean-flow conditions for OH, MH, and PH salinity zones in recent years (2011 to 2015) to proposed criteria, revealing significant differences among properties with respect to attaining putative targets (Tables 4–6). Spring *chl-a* for the OH salinity zone in recent years exceeded proposed criteria based on all data, the 1960s (bold type), or previous analyses, with percent differences from 19.6 to 86.6% (Table 4). Conversely, summer and fall *chl-a* for the OH salinity zone in recent years were lower than proposed criteria on several time bases, and thereby in compliance, with percent differences from −4.97 to −26.7% (Table 4). Seasonal *chl-a* for the MH salinity zone in recent years exceeded proposed criteria based on all data, the 1960s (except fall), or previous analyses, especially in spring with percent differences from 32.7 to 346% (Table 4). Lastly, seasonal *chl-a* concentrations for the PH salinity zone in recent years were similar to proposed criteria based on all data, but higher than criteria based on the 1960s (except fall) or previous analyses (Table 4). These findings suggest further reductions of *chl-a* in spring will be required to meet proposed criteria for the OH salinity zone, and in all seasons for MH and PH salinity zones, depending on the time bases used to set criteria.

Model predictions of Secchi depth for OH, MH, and PH salinity zones in mean-flow conditions were generally lower (=decreased water clarity) in recent years (2011 to 2015) than proposed criteria based on all data, the 1960s (bold type), or 1985 to 1989 (Table 5). An exception was Secchi depth for the OH salinity zone in spring that was slightly higher in recent years (=increased water clarity), ostensibly associated with a downward trajectory of *chl-a* in the upper bay following a ban of PO_4_^3−^ in detergents. Model predictions for the MH salinity zone in mean-flow conditions showed lower Secchi depth (=decreased water clarity) in recent years than proposed criteria based on all data, the 1960s (bold type), or 1985 to 1989, with percent differences from 5.25 to 30.5%. Consistent with the MH salinity zone, model predictions of Secchi depth for the PH salinity zone in mean-flow conditions were generally lower (=decreased water clarity) in recent years than proposed criteria based on all data, the 1960s (bold type), or 1985 to 1989, with percent differences from 3.77 to 22.0%.

Lastly, model predictions of NO_2_ + NO_3_ for the OH salinity zone in mean-flow conditions in recent years (2011 to 2015) consistently exceeded proposed criteria based on all data, the 1960s, or low-flow conditions in recent years (bold type) (Table 6). Conversely, model predictions of NO_2_ + NO_3_ for MH and PH salinity zones in low-flow conditions in recent years met proposed criteria based on all data or the 1960s. These findings were consistent with decreased TN and NO_2_ + NO_3_ loading after 1980^15–18^, and increased nutrient consumption accompanying a historical increase of *chl-a* in the bay^9,17,18^. Model predictions of NO_2_ + NO_3_ for MH and PH salinity zones in low-flow conditions in recent years were consistently lower than those in mean-flow conditions, capturing climatic effects on TN and NO_2_ + NO_3_ loading, shown as positive percent differences (Table 6). These model predictions guided numerical criteria as they accounted for the downward trend of annual TN and NO_2_ + NO_3_ loading since 1980, and for climatic effects on TN and NO_2_ + NO_3_ loading. Basing proposed criteria for NO_2_ + NO_3_ on model predictions in low-flow conditions in recent years proved effective for the OH salinity zone, but seasonal depletion of NO_2_ + NO_3_ in summer and fall limited the usefulness of this approach for MH and PH salinity zones. A continuing increase of model predictions of *chl-a* for the MH salinity zone in mean-flow conditions may explain the decrease of NO_2_ + NO_3_, i.e., increased consumption by phytoplankton, suggesting that progress should not be defined by a single water-quality property.

### Long-term trends of water-quality properties

**Figure 6:**
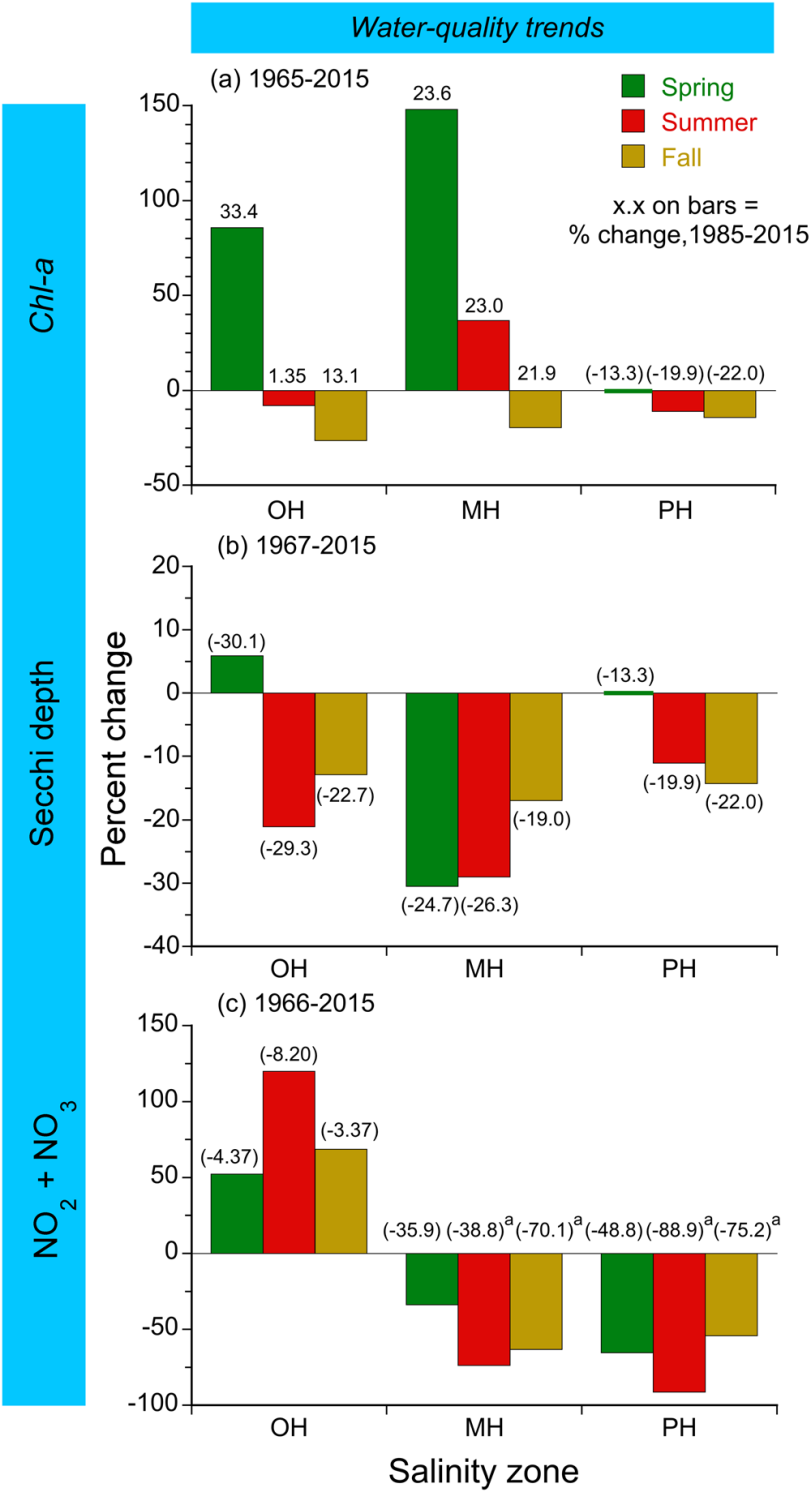
(**a**–**c**) Long-term trends of flow-adjusted *chl-a* (geometric mean), Secchi depth, and NO_2_ + NO_3_ for OH, MH, and PH salinity zones in the main-stem bay. Percent changes represent differences over time for model predictions in mean-flow conditions. Superscript a denotes summer NO_2_ + NO_3_ trends when observed values from 2011 to 2015 were substituted for flow-adjusted values as model predictions in mean-flow conditions were zero.

Model predictions of water-quality properties in mean-flow conditions were used to quantify trends of *chl-a*, Secchi depth, and NO_2_ + NO_3_ as percent changes (Fig. 6a–c). Long-term trends of *chl-a* differed by salinity zone and season. Spring *chl-a* showed increasing trends for OH and MH salinity zones, but was essentially constant for the PH salinity zone; summer *chl-a* showed an increasing trend for the MH salinity zone, but decreasing trends for OH and PH salinity zones; fall *chl-a* showed consistent, decreasing trends for OH, MH, and PH salinity zones (Fig. 6a). Percent changes from 1985 to 2015 placed at the top of individual bars showed that recent trends of *chl-a* sometimes differed from the complete time series (compare Fig. 4-c). Examples include *chl-a* for the OH salinity zone in summer and fall with decreasing trends from 1965 to 2015, but increasing trends from 1985 to 2015; the MH salinity zone in fall with a decreasing trend from 1965 to 2015, but an increasing trend from 1985 to 2015; the PH salinity zone in spring with a decreasing trend from 1985 to 2015, but nearly constant *chl-*a for the complete time series.

Secchi depth showed consistent, decreasing trends for OH, MH, and PH salinity zones from 1967 to 2015 as percent changes, with the exception of the OH salinity zone that showed an increasing trend in spring (Fig. 6b). Percent changes of Secchi depth from 1985 to 2015 placed at the top of individual bars showed decreasing trends from −13.3 to −30.1% for OH, MH, and PH salinity zones in all seasons.

NO_2_ + NO_3_ for the OH salinity zone showed increasing trends in all seasons from 1966 to 2015 as percent changes (Fig. 6c). A reversal of sign for percent changes from 1985 to 2015 occurred for the OH salinity zone shown at the top of individual bars. Percent changes for MH and PH salinity zones from 1966 to 2015 showed consistent, decreasing trends of NO_2_ + NO_3_ that continued throughout the time series.

### Trajectories of *chl-a* vs TN loading

**Figure 7:**
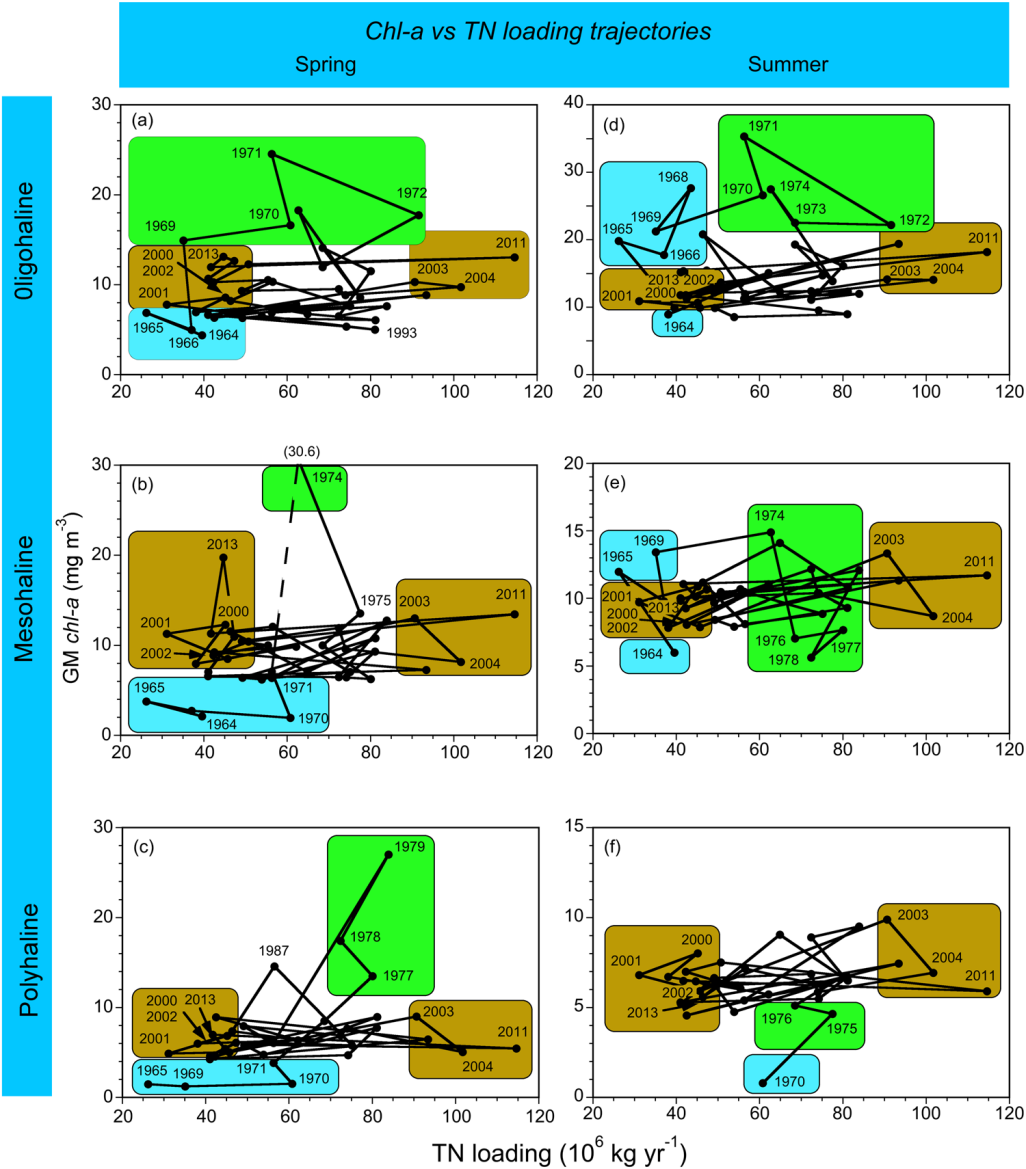
(**a**–**f**) Trajectories of observed spring and summer *chl-a* (geometric means) vs annual TN loading (10^6^ kg yr^−1^) for OH, MH, and PH salinity zones in the main-stem bay. Color-shaded polygons distinguish data for the 1960s (blue), 1970s (green), and 2000s (amber). Data for the 2000s indicate climatic effects on TN loading did not lead to commensurate *chl-a* responses.

Long-term data on *chl-a* were paired with annual TN loading to depict trajectories by salinity zone and season (Fig. 7a–f). Data for the OH salinity zone from 1964 to 2015 provided strong evidence of shifted trajectories of *chl-a* vs TN loading in spring and summer, consisting of higher *chl-a* in years with mid-range TN loading (1970, 1971, 1973, 1974) than in recent years (2003, 2004, 2011) characterized by the highest annual TN loading in the time series (Fig. 7a,d). Much higher spring *chl-a* from 17–25 mg m^−3^ and summer *chl-a* from 22–35 mg m^−3^ occurred at TN loading of 60–80 (×10^6^) kg yr^−1^ in the early 1970s, than spring *chl-a* from 10–13 mg m^−3^ and summer *chl-a* from 14–18 mg m^−3^ at higher TN loading >90 (×10^6^) kg yr^−1^ in the 2000s (2003, 2004, 2011). Spring and summer *chl-a* in recent years with TN loading <50 (×10^6^) kg yr^−1^ (2000–2002, 2013) were similar to *chl-a* at much higher TN loading in the 2000s (2003, 2004, 2011).

The trajectory of *chl-a* vs TN loading in spring for the MH salinity zone showed a weaker response of *chl-a* to TN loading than for the OH salinity zone (compare Fig. 7a,b). Two prominent spring *chl-a* maxima from 20–31 mg m^−3^ for the MH salinity zone occurred at moderate TN loading of 45–65 (×10^6^) kg yr^−1^ in 1974 and 2013, contrasted with much lower *chl-a* from 2–7 mg m^−3^ at similar TN loading from 1964 to 1971 (Fig. 7b). The trajectory of *chl-a* vs TN loading in summer for the MH salinity zone showed *chl-a* was less sensitive to changes of TN loading than in spring, with *chl-a* from 6–15 mg m^−3^ at low to moderate TN loading of 25–60 (×10^6^) kg yr^−1^ between 1964 and 1974 (compare Fig. 7b,e). Summer *chl-a* in this range was similar to *chl-a* from 9–13 mg m^−3^ at higher TN loading >90 (×10^6^) kg yr^−1^ in the 2000s (2003, 2004, 2011) (Fig. 7e).

Lastly, the trajectory of *chl-a* vs TN loading in spring for the PH salinity zone showed low *chl-a* from 2–5 mg m^−3^ at low to moderate TN loading of 25–60 (×10^6^) kg yr^−1^ from 1965–1974, higher *chl-a* from 9–27 mg m^−3^ at moderate to high TN loading of 70–90 (×10^6^) kg yr^−1^ in the late 1970s, and much lower *chl-a* from 5–9 mg m^−3^ in the 2000s (2003, 2004, 2011) at higher TN loading >90 (×10^6^) kg yr^−1^ (Fig. 7c). Summer *chl-a* ranged from ~1–5 mg m^−3^ for the PH salinity zone at moderate TN loading of 60–80 (×10^6^) kg yr^−1^ in the early- to mid-1970s, lower than *chl-a* from 6–10 mg m^−3^ at higher TN loading >90 (×10^6^) kg yr^−1^ in the 2000s (2003, 2004, 2011) (Fig. 7f). Spring and summer *chl-a* for the PH salinity zone in recent years with low TN loading <50 (×10^6^) kg yr^−1^ (2000–2002, 2013) were similar to *chl-a* at much higher TN loading in the 2000s (2003, 2004, 2011) (Fig. 7c,f).

### Water-quality criteria for major tributaries

**Figure 8:**
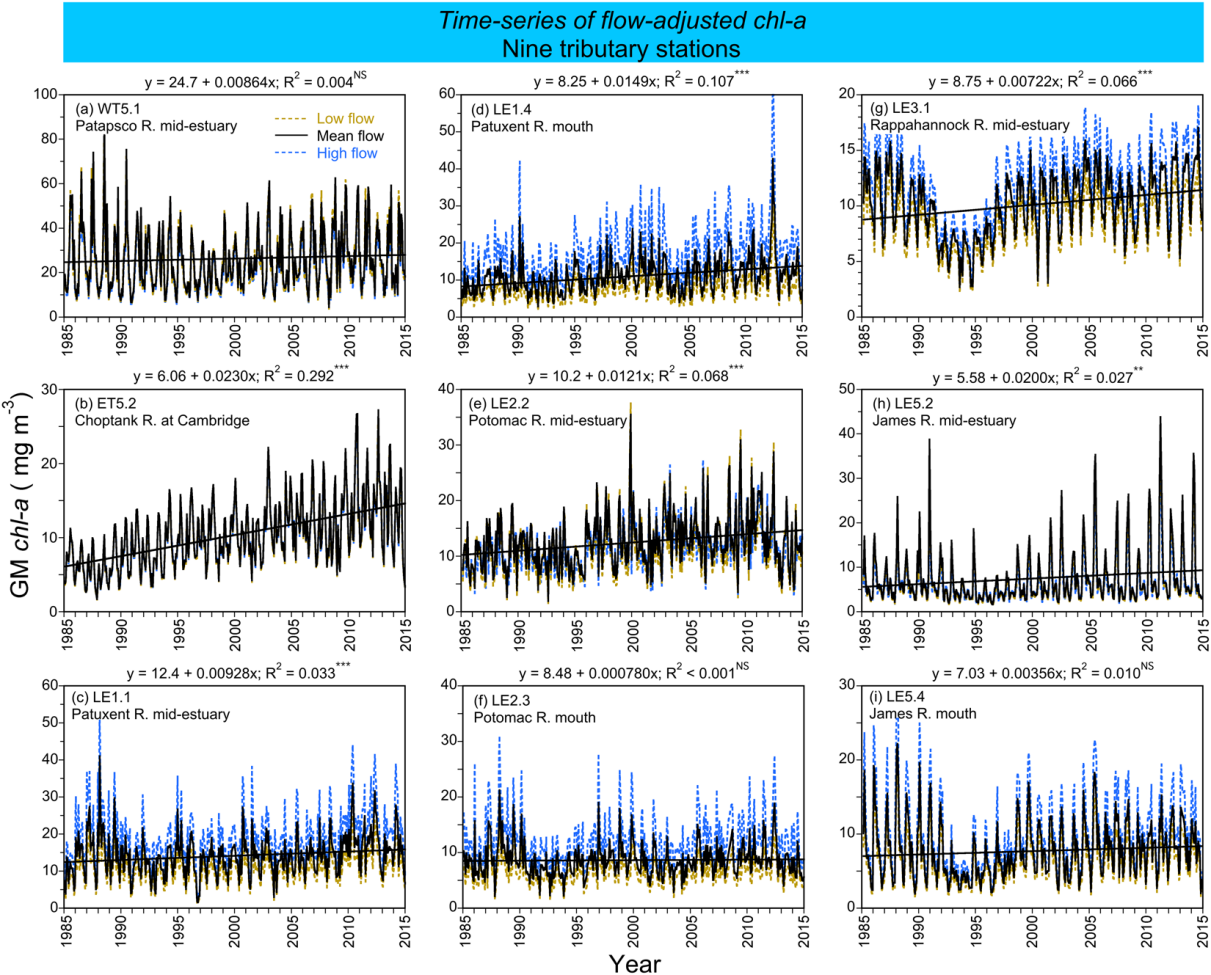
(**a**–**i**) Time series of flow-adjusted model predictions of mean, monthly *chl-a* (geometric mean) at tidal stations in nine tributaries of Chesapeake Bay from 1985 to 2015. *Crosses* show observed values; *solid black lines* depict model predictions in mean-flow conditions; *dashed blue lines* show predictions in high-flow (=low-salinity) conditions; *dashed brown lines* show predictions in low-flow (=high-salinity) conditions.

Models for major tributaries in the bay were based on means, 10^th^, and 90^th^ percentiles of salinity at each station as surrogates for flow to capture seasonal to inter-annual variability. Time series of salinity-adjusted model predictions of *chl-a* at nine tributary stations from 1985 to 2015 resembled analogous predictions for the main-stem bay (Fig. 8a–i). Model predictions in mean-salinity conditions showed increasing *chl-a* from 1985 to 2015 at the mouths of the Patuxent River (Fig. 8d) and James River (Fig. 8i), and at mid-estuary stations in the Choptank River (Fig. 8b), Potomac River (Fig. 8e), Rappahannock River (Fig. 8g), and James River (Fig. 8h). These long-term trends of *chl-a* were similar to those for MH and PH salinity zones in the main-stem bay (compare Fig. 4b,c). Observed vs model-fitted values of *chl-a*, Secchi depth, and NO_2_ + NO_3_ for nine tributary stations, and time series of salinity-adjusted model predictions of Secchi depth and NO_2_ + NO_3_ complementing analogous predictions of *chl-a* (Fig. 8a–i) are presented in Supplementary Material.

**Figure 9:**
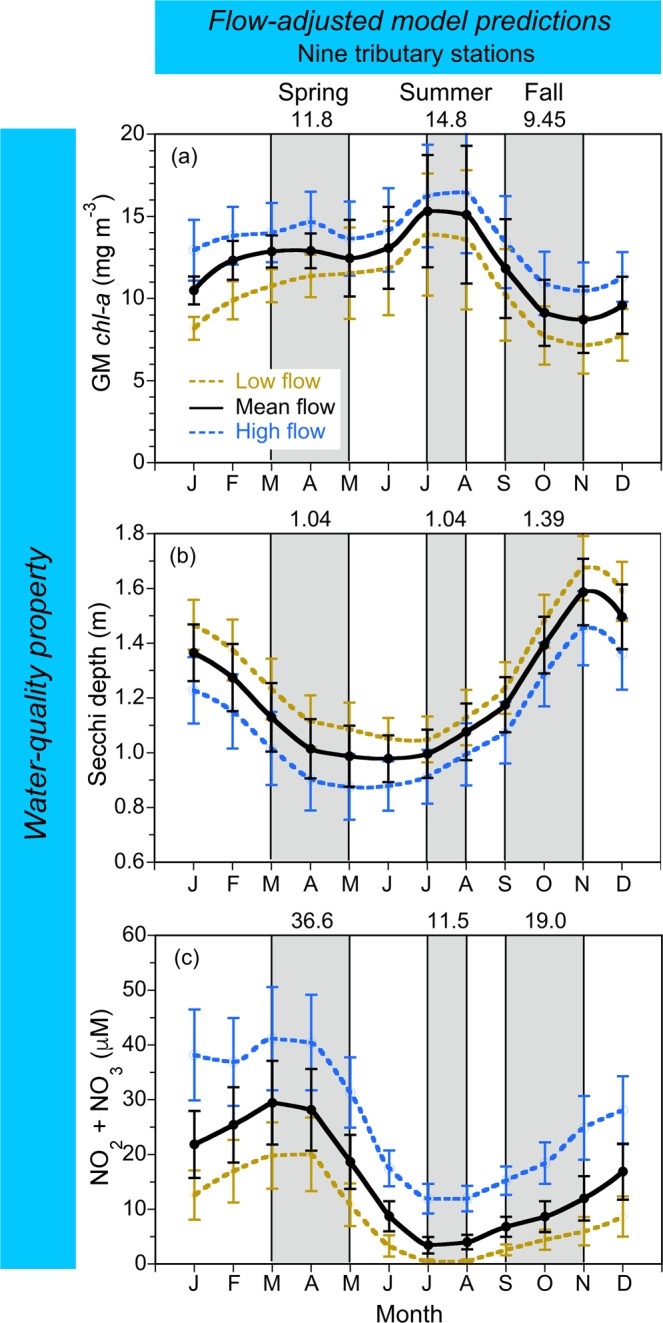
(**a**–**c**) Mean, monthly flow-adjusted model predictions of *chl-a* (geometric mean), Secchi depth, and NO_2_ + NO_3_ aggregated from long-term data for nine tributary stations in Chesapeake Bay from 1985 to 2015. *Crosses* show observed values; *solid black lines* depict model predictions in mean-flow conditions; *dashed blue lines* show predictions in high-flow (=low-salinity) conditions; *dashed brown lines* show predictions in low-flow (=high-salinity) conditions. Error bars are ±1 standard error (SE) of the estimates. Vertical shaded areas denote spring, summer and fall, with seasonal means depicted on each panel.

Numerical criteria for *chl-a*, Secchi depth, and NO_2_ + NO_3_ for nine tributary stations were based on salinity-adjusted model predictions (Fig. 8a–i; Supplementary Material, Figs S3, S5) aggregated at a monthly scale (Fig. 9a–c). Model predictions of *chl-a* in mean-salinity conditions ranged from 9.45–14.8 mg m^−3^ (Fig. 9a), compared to 5.36–14.7 mg m^−3^ for OH, MH, and PH salinity zones in the main-stem bay (Fig. 5a–c). Model predictions of Secchi depth in mean-salinity conditions ranged from 1.04–1.39 m at tributary stations (Fig. 9b), compared to 0.87–1.77 m for salinity zones in the main-stem bay (Fig. 5d–f). Lastly, model predictions of NO_2_ + NO_3_ in mean-salinity conditions ranged from 3.74–25.5 μM at tributary stations (Fig. 9c), compared to 0.690–57.4 μM for the main-stem bay (Fig. 5g–i). These proposed criteria for tributaries were similar to those for the main-stem bay.

## Discussion

### Climatic effects on phytoplankton

An essential starting point in developing numerical water-quality criteria in the studies described here was to adjust for climatic effects. This allowed us to take account of spatio-temporal variability imposed by climatic effects, to distinguish long-term trends reflecting anthropogenic eutrophication, and to set realistic targets for restoration. Climatic effects on phytoplankton dynamics have been well described in estuarine-coastal ecosystems using long-term data from shipboard, aircraft, and satellite measurements^26–35^. Previous studies contributed to this understanding of climatic effects on nutrient loading, *chl-a*, floral composition, and NPP in Chesapeake Bay^17–19,24,33^ and the Neuse and New River estuaries^34^.

A logical sequence emerged from these studies, accentuating hydrological regulation of TN and NO_2_ + NO_3_ loading with predictable consequences for water-quality properties and phytoplankton dynamics in the bay. Summarizing, low-flow, “dry” conditions lead to a landward shift of N-limitation toward OH and MH salinity zones, resulting in lower *chl-a*, lower NPP, and a decreased proportion of diatoms in the flora; conversely, high-flow, “wet” conditions extend the area of N sufficiency seaward to MH and PH salinity zones, resulting in higher *chl-a*, higher NPP, and an increased proportion of diatoms in the flora^17–19,24,33^. Dissolved and suspended materials affecting Secchi depth are similarly sensitive to climatic effects, with higher inputs of bio-optically active constituents in high-flow, “wet” conditions than in low-flow, “dry” conditions^17,18^.

We applied this logic to develop numerical water-quality criteria for the bay, using predictions conditioned on specific model inputs of flow and salinity to distinguish long-term trends from spatio-temporal variability imposed by climatic effects^17,18^. SRF and frequencies of predominant weather patterns identified “dry” and “wet” conditions (Fig. 2)^19–21^; salinity served as an explanatory variable in all statistical models, and as a proxy for freshwater flow at tributary stations. Time series of flow-adjusted model predictions of *chl-a*, Secchi depth, and NO_2_ + NO_3_ (Fig. 4a–i), and predictions aggregated at a monthly scale (Fig. 5a–i), documented climatic effects consistent with our previous studies based on annual means^17,18^.

### Numerical water-quality criteria

Statistical models used to generate flow-adjusted predictions of *chl-a*, Secchi depth, and NO_2_ + NO_3_ allowed us: (1) to derive numerical water-quality criteria by season and salinity zone; (2) to adjust for climatic effects in establishing these criteria; (3) to propose criteria corresponding to climatic conditions reflecting decreased TN and NO_2_ + NO_3_ loading (MH and PH salinity zones) or increased light limitation (OH salinity zone); (4) to evaluate attainment by comparing recent values of water-quality properties to criteria based on selected time periods.

Multiple lines of scientific evidence, consisting of a historical increase of *chl-a*, low dissolved oxygen (DO), decreased water clarity, and harmful algal blooms (HAB), were used previously to develop numerical *chl-a* criteria for Chesapeake Bay^25^. We were guided by a “protective” approach to avoid impairments associated with nutrient over-enrichment and high *chl-a*. Sutula *et al*.^36,37^ recently developed *chl-a* criteria for the San Francisco Bay estuary (SFB) using a similar approach designed to lessen the probability of ecosystem impairments such as HAB and low DO.

Numerical *chl-a* criteria developed in previous studies on Chesapeake Bay used a different statistical approach than we used here. We designated the 1960s as a “reference period” and computed geometric means and 90th percentiles for a period when symptoms of anthropogenic eutrophication were less evident^25^. Those studies proposed *chl-a* criteria for spring and summer with means from 1.4–15 mg m^**−**3^ as goals, and 90^th^ percentiles from 4.3–45 mg m^**−**3^ as thresholds. We later concluded that deriving numerical *chl-a* criteria with the 1960s as a reference period hinged on the validity of an assumption that water quality was better during that time. Although an absence of data for pristine conditions limited our options for a reference period, we believe that using the 1960s was too simple as: (1) mean, annual *chl-a* was higher for the OH salinity zone in the 1960s than in the mid- to late-1970s; (2) mean, annual *chl-a* was lower for MH and PH salinity zones in the 1960s, coinciding with persistent low-flow, “dry” conditions; (3) maxima of mean, annual *chl-a* for MH and PH salinity zones occurred by the mid-1980s following a decade of high flow, “wet” conditions; (4) spatio-temporal variability of mean, annual *chl-a* from 1995 to 2015 was driven by irregular “dry” and “wet” conditions. These several observations accentuated the need to adjust for climatic effects^17,18^ rather than to base criteria on the 1960s as a reference period.

To avoid limitations of using the 1960s as a reference period, our new analyses combined historical and monitoring data (1960s to 2015) to derive numerical criteria, focusing on climatic effects and long-term trends of water-quality properties. We based these analyses on data aggregated at monthly to seasonal scales, expanded the set of response variables to include *chl-a*, Secchi depth as a measure of water clarity, and NO_2_ + NO_3_ concentrations as a measure of nutrient over-enrichment, and considered reference periods specific to each water-quality property (Tables 4–6). Comparative data for nine tributary stations also supported criteria for *chl-a*, Secchi depth, and NO_2_ + NO_3_ revealing similar ranges for the main-stem bay and tributaries (Figs 5a–i and 9a–c, and Supplementary Material).

**Figure 10:**
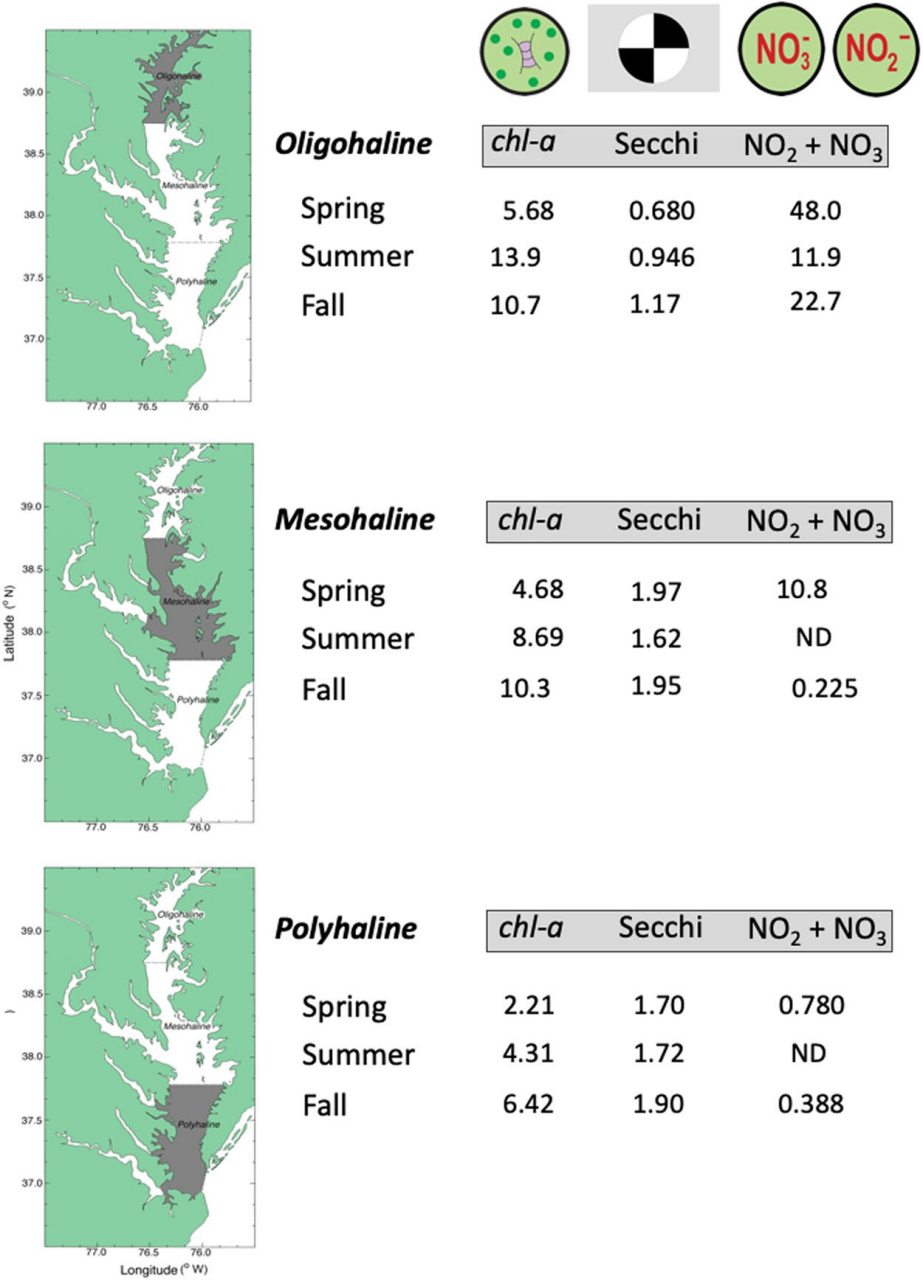
Conceptual diagram summarizing proposed water-quality criteria for Chesapeake Bay. Symbols courtesy author Tracey Saxby, Integration and Application Network, University of Maryland Center for Environmental Science (ian.umces.edu/imagelibrary/).

Proposed numerical criteria for *chl-a*, Secchi depth, and NO_2_ + NO_3_ presented in Tables 4–6 consist of specific values and their underlying bases. A conceptual diagram summarizes these criteria for the main-stem bay, providing a simple view based on salinity zone, season, and water-quality property (Fig. 10). These criteria improve upon earlier analyses by accounting for climatic effects and long-term trends of water-quality properties on seasonal and spatial bases.

### Long-term trends, current status, and transitions

Numerical water-quality criteria have practical applications to assess long-term trends, current status, and transitions. We previously reported a significant, decreasing trend of *chl-a* for the OH salinity zone after the mid-1960s using model predictions in mean-flow conditions for data aggregated at an annual scale. This trend was ascribed to a ban on PO_4_^3−^ that enhanced P-limitation of phytoplankton in the upper bay^23^, leading to increased throughput of TN and NO_2_ + NO_3_ to MH and PH salinity zones^17,18^. New analyses of long-term trends suggest large reductions of TN and NO_2_ + NO_3_ loading in spring will be required to attain proposed *chl-a* criteria for OH, MH, and PH salinity zones (Table 4). A continuing, upward trend of *chl-a* for the MH salinity zone is especially problematic in the context of summertime hypoxia in the bay^3,16^.

Bio-optical conditions as Secchi depth have deteriorated throughout the bay, as shown by generally decreasing trends of model predictions for OH, MH, and PH salinity zones in mean-flow conditions from 1967 to 2015 (Fig. 6b). The largest percent changes of Secchi depth occurred for the MH salinity zone from 1967 to 2015, matched by trends of similar magnitude from 1985 to 2015. Some of the long-term trend of Secchi depth for the MH salinity zone can be explained by increased *chl-a* (Fig. 6a), but increased phytoplankton biomass does not account for decreasing trends of Secchi depth in the PH salinity zone (Fig. 6b) where *chl-a* has remained essentially constant from 1985 to 2015 (Fig. 6a).

Our analyses suggest further reductions of TN and NO_2_ + NO_3_ loading will be required to attain *chl-a* criteria that constrain the magnitude and extent of the spring diatom bloom, particularly in mean-flow and high-flow years (Tables 4, 6). An important review of phytoplankton dynamics in the bay described NO_2_ + NO_3_ loading and density stratification associated with the spring freshet of the Susquehanna River as essential triggers of the spring bloom^35^. Consecutive features of the annual phytoplankton cycle include seasonal exhaustion of nutrients by the spring bloom, landward migration of the *chl-a* maximum, subsequent deposition of diatom biomass, and persistent thermal stratification in summer, culminating in deep-water DO depletion, and coinciding with a summer NPP maximum driven by N regeneration^18^. Decreasing trends of NO_2_ + NO_3_ for MH and PH salinity zones expressed as negative percent changes from 1985 to 2015 represent encouraging, albeit modest progress that is consistent with decreased TN and NO_2_ + NO_3_ loading since the early 1980s. Unfortunately, continued increases of model predictions of *chl-a* for the MH salinity zone in mean-flow conditions suggest recent progress to reduce TN and NO_2_ + NO_3_ loading has not been sufficient to lower phytoplankton biomass in the mid-bay (Fig. 4b), reversing eutrophication of the past half century^17,18^.

### Trajectories of *chl-a* vs TN loading

Pronounced decadal differences in trajectories of *chl-a* vs TN loading expressed climatic effects and anthropogenic eutrophication in the bay^17^, exemplified by low *chl-a* per unit TN in the 1960s, high *chl-a* per unit TN in the 1970s, and relatively stable *chl-a* regardless of TN loading in the 2000s (Fig. 7a–f). These differences coincided with contrasting climatic conditions in the 1960s (“dry”) and 1970s (“wet”), a doubling of flow-adjusted TN and NO_2_ + NO_3_ loading from 1945 to 1980^17^, decreased ratios of *chl-a* : TN from 1984 to 1992, and increased ratios in mean-flow conditions after 1994^17,18^. Notable features of trajectories included *chl-a* ~20 mg m^−3^ for the MH salinity zone that coincided with TN loading ~45 (×10^6^) kg yr^−1^ in 2013, compared to *chl-a* < 6 mg m^−3^ at TN loading of 25–60 (×10^6^) kg yr^−1^ in the mid-1960s to early 1970s (Fig. 7b). Similarly, *chl-a* ~15 mg m^−3^ for the PH salinity zone occurred at TN loading ~60 (×10^6^) kg yr^−1^ in 1987, compared to *chl-a* < 4 mg m^−3^ at slightly higher TN loading in 1970 (Fig. 7c).

Trajectories of *chl-a* vs TN captured near-term climatic effects for the 2000s (Fig. 7a–f, amber shading). These data showed similar *chl-a* in “wet” years (e.g. 2003, 2004, 2011) with highest TN loading in the time series, and “dry” years (e.g. 2000–2002, 2013) with low TN loading similar to the 1960s. Thus, *chl-a* in recent years with reduced TN loading did not decrease to earlier, lower concentrations. These findings are consistent with the concept of “Return to Neverland” put forth by Duarte *et al*.^38^, postulating that a simple retracing of past trajectories is unlikely following a reversal of anthropogenic eutrophication. “Neverland”^38^ was based on three-year means of *chl-a* vs TN loading, while trajectories of *chl-a* vs TN loading presented here were based on flow-adjusted annual means (Fig. 7a–f). Nonetheless, the non-linear responses of *chl-a* to reduced TN loading we observed in recent years support the applicability of this concept to the bay. Non-linear trajectories have been explained previously by long-term changes of ecosystem structure, including regeneration of legacy nutrients, changes of the cell-size distribution of phytoplankton, and altered floral composition^38,39^. Evidence for causes of non-linear responses of *chl-a* to TN loading in the bay consists of: (1) bio-optical properties with a change in coupling constants of Secchi depth and K_D(PAR)_ that suggest the particle-size distribution may have shifted over time^40,41^; (2) long-term trends in the cell-size distribution and floral composition of phytoplankton from 1985 to 2007^24^.

### Case studies: comparisons with other ecosystems

Anthropogenic eutrophication of estuarine-coastal ecosystems around the world has been extensively studied from the perspective of remediation. Analyses centered on two approaches to reverse effects of nutrient over-enrichment, “corrective” and “protective”. The following paragraphs give examples of how these approaches have been implemented in several at-risk ecosystems with comparisons to Chesapeake Bay.

Novel statistical methods were used by Sutula *et al*. to develop *chl-a* criteria for SFB directed at lessening the likelihood of impairments^36,37^. SFB is characterized by high nutrient concentrations but has yet to experience deleterious impacts commonly associated with anthropogenic eutrophication. SFB has a shorter residence time than Chesapeake Bay, possibly explaining why potential impairments have yet to appear. A significant ecosystem shift occurred in SFB following introduction of the Asian clam, *Potamocorbula amurensis*, leading to increased benthic grazing that significantly reduced phytoplankton biomass in the northern estuary^12^. Some regions have not been affected by this invasive species, and resistance to nutrient over-enrichment elsewhere in SFB appears to be weakening, supported by observations of: (1) a three-fold increase of *chl-a* in the south bay during summer and fall since 1999^42^; (2) common occurrences of HAB taxa throughout the estuary^43–45^; (3) increased incidences of hypoxia with DO < 5 mg L^−1^ in the southernmost bay^46^.

These worrisome changes in SFB stimulated the development of *chl-a* thresholds intended to be “protective” from impairments, distinct from Chesapeake Bay where “corrective” measures are directed at reversing existing impairments. Significant relationships of *chl-a* to HAB abundance and low DO derived from quantile regressions indicated increased *chl-a* leads to an increased risk of impairments. Conditional probability analysis identified a *chl-a* threshold of 13 mg m^−3^, below which probabilities of exceeding alert levels for HAB abundance and toxins decreased. A similar *chl-a* threshold of 13 to 16 mg m^−3^ was linked to a mandated water-quality criterion of 80% saturation for DO. Higher *chl-a* thresholds from 25–40 mg m^−3^ corresponded to 0.5 probability of exceeding alert levels for HAB abundance and DO < 5.0 mg L^**−**1^ in southerly regions of SFB. While these predictive relationships between *chl-a*, HAB, and low DO were sensitive to climatic effects and highly variable, “protective” *chl-a* thresholds provided starting points for SFB based on potential impairments^36,37^. Similar to SFB, our previous analyses to develop numerical *chl-a* criteria for Chesapeake Bay were based on multiple lines of scientific evidence, focusing on long-term trends and ecosystem impairments. It is noteworthy that comparable thresholds emerged for SFB^36,37^ and Chesapeake Bay^25^, pointing to the merits of a “protective” approach to avoid negative consequences of anthropogenic eutrophication.

The Potomac River (PR) is the largest tributary of Chesapeake Bay. PR has been studied extensively since the 1960s following dense algal blooms that were stimulated by nutrient over-enrichment. PR is representative of mid-Atlantic estuaries in the United States that have experienced moderate to high levels of degradation. Riverine parts of PR have undergone extensive hydrologic modifications in the Washington, D.C. urban area. The large sub-estuary drains an extensive watershed covering several states and is highly responsive to climatic effects, such as droughts and floods, that influence the entire region. Long-term studies of PR by Jaworski *et al*.^47^ and a synthesis by Bricker *et al*.^48^ documented significant impacts of anthropogenic eutrophication, despite decreasing trends of N loading from the upper river basin and declining concentrations of NO_3_ in surface waters. To this point, Jaworski *et al*.^47^ estimated a 50% reduction of 1985 base-year TN loading, including 54–65% reductions of non-point sources and continued reductions of TN in wastewater effluent, would be required to meet water-quality criteria for PR. Reductions of TN loading of similar magnitude have been recommended for Chesapeake Bay, but progress to attain this goal has been modest. Other approaches in PR such as shellfish aquaculture have been proposed to complement land-based measures to decrease TN loading, but oyster restoration alone is deemed unlikely to reverse symptoms of anthropogenic eutrophication in either the sub-estuary^48^ or the main-stem bay^49^.

In northern Europe, extensive management efforts have been directed at Danish coastal waters to reverse deleterious symptoms of anthropogenic eutrophication. Widespread hypoxia in the Danish straits stimulated the 1985 NPo Action Plan, and long-term studies chronicled successful reductions of nutrient loading^13,38,39,50,51^. Riemann *et al*.^13^ described 25 years of water-quality responses in a “corrective” approach that has produced ~50% decreases of N and P loading since 1990. These dramatic decreases of N and P loading led to decreased nutrient concentrations in receiving waters, a modest decrease of *chl-a*, and restoration of macro-algae to deep waters. Notably, the decrease of *chl-a* has failed to match nutrient reductions stoichiometrically^13^, consistent with observations for other estuarine-coastal ecosystems detailed by Duarte *et al*.^38^ and Carstensen *et al*.^39^ Nonetheless, successful corrective actions in Danish coastal waters give hope that significant reductions of N and P inputs may reverse deleterious symptoms of anthropogenic eutrophication in other estuarine-coastal ecosystems. A key difference between Danish coastal waters and Chesapeake Bay is that decreasing trends of nutrients in the N. European ecosystem entailed changes in the relationships of nutrient loading to freshwater flow^50,51^, while relationships of TN and NO_2_ + NO_3_ loading to SRF in the bay have not returned to previous conditions^17,18^.

### Expectations, restoration, possibilities

How might we use climatic effects on water-quality properties to develop numerical criteria that inform us about targets for restoration? We submit that flow-adjusted model predictions constitute guidance to identify attainable values for water-quality properties by incorporating climatic effects. In this way, numerical criteria for *chl-a*, Secchi depth, and NO_2_ + NO_3_ bracket realistic, pragmatic goals that can be compared to current conditions to gauge progress. Too often, bay-health assessments have ignored spatio-temporal variability associated with climatic effects. Our analysis of long-term data accentuates the sensitivity of water-quality properties to climatic effects, showing that assessments based on one or several years can be misleading. To this point, claims of progress toward improved water quality following low-flow, “dry” conditions are generally premature or erroneous. An apt analogy would be skepticism about “global warming” based on periodic cold winters experienced in a historical context of rising temperatures.

A shallow trajectory toward reduced TN and NO_2_ + NO_3_ loading in the bay^17,18^ has been accompanied by troubling trajectories of *chl-a* vs TN loading (Fig. 7a–f), confirming that a simple return to previous conditions may prove elusive^38^. An increasing trend of model predictions of *chl-a* for the MH salinity zone in mean-flow conditions (Fig. 4b) provides worrisome evidence that organic matter derived from phytoplankton has not responded to decreased TN and NO_2_ + NO_3_ loading in the region where annual hypoxia/anoxia occurs^3,16–18^^,^^24,25^. Successful efforts in Danish coastal waters produced significant, 50% reductions of nutrient inputs and changed relationships between nutrient concentrations and flow^50,51^. In contrast, modest progress to reduce nutrient inputs in the bay has consisted of <20% reductions of TN and NO_2_ + NO_3_ loading, and relationships between loading and SRF have not returned to previous conditions^17^. Absent such a change, climatic effects will continue to dominate seasonal to inter-annual variability of TN and NO_2_ + NO_3_ loading, and a “corrective” approach alone is unlikely to yield significant improvements of water quality. We suggest that specifying criteria based on sustained adherence to flow-adjusted model predictions represents an approach that takes advantage of time-series data on water-quality properties, while incorporating climatic effects that strongly influence contemporary conditions.

## Conclusions

Numerical water-quality criteria were developed for Chesapeake Bay using statistical models to adjust for climatic effects. These criteria were directed at a “corrective” approach, setting proposed criteria in a domain of the reasonable, based on long-term observations. Summarizing:

- Spatio-temporal variability of water-quality properties exemplified by *chl-a*, Secchi depth, and NO_2_ + NO_3_ is driven primarily by climatic effects, superimposed on long-term trends associated with anthropogenic eutrophication;
- Flow-adjusted TN and NO_2_ + NO_3_ loading to the bay captures the course of anthropogenic eutrophication since World War II, providing a rationale to adjust time series of water-quality properties for climatic effects;
- Statistical models applied to time series of *chl-a*, Secchi depth, and NO_2_ + NO_3_ supported numerical water-quality criteria for the main-stem bay and major tributaries;
- Flow-adjusted model predictions were used to compute long-term trends of water-quality properties, to specify numerical criteria constituting realistic goals, and to assess progress toward attainment using comparisons with conditions in recent years;
- This ‘corrective’ approach based on numerical criteria extends work on other estuarine-coastal ecosystems to incorporate climatic effects, thereby addressing spatio-temporal variability, resolving long-term trends, and quantifying improvements.

## Methods

### Study site

The focus of these studies was Chesapeake Bay in the mid-Atlantic region of the United States. The bay is a shallow, partially mixed, temperate estuary of the Susquehanna River, with a main-stem surface area ~8,000 km^2^, receiving inputs of freshwater, sediment, and solutes from an extensive 165,000 km^2^ watershed. North-south gradients of salinity, nutrients, and light penetration characterize the ecosystem, with a number of significant tributaries also contributing freshwater and solutes. These tributaries include the Patapsco, Patuxent, Potomac, Rappahannock, York, and James Rivers on the western shore, and the Choptank, Pocomoke, and Nanticoke Rivers on the eastern shore. A map showing major geographic features, salinity zones, and station locations (Fig. 1) was produced with Surfer v. 8 (Golden Software) and customized with Adobe Photoshop v. CS6.

### Data sources

Long-term data on water-quality properties for the bay and tributaries from 1960 to 2015 supported this work. Discharge records of daily freshwater flow (ft^3^ d^−1^) from the Susquehanna River gaging station at Conowingo Dam were obtained from the United States Geological Survey (USGS) Non-tidal Monitoring Program http://cbrim.er.usgs.gov/ ^52^, converted to metric units (m^3^ d^−1^), and used to compute mean, monthly (SRF) and cumulative, monthly (SUM) flow. N loading (TN, NO_2_ + NO_3_) was obtained from the same USGS source. SRF and SUM were log_10_-transformed for normalcy and used as predictor variables in GAM. Mann-Kendall tests in the “R” package ‘wq’ revealed no significant trend of SRF for the period corresponding to water-quality observations^53^. Availability of water-quality data analyzed in this paper is assured via the data hub of the U.S. Environmental Protection Agency, Chesapeake Bay Program (CBP), Annapolis, Maryland https://www.chesa-peakebay.net/what/data.

Water-quality properties consisted of *chl-a* (mg m^−3^), Secchi depth (m), NO_2_ + NO_3_ (μM), salinity, and temperature (°C) for the surface mixing layer. Data sources included historical observations from the Chesapeake Bay Institute and monitoring data from CBP^54–56^. Sampling stations for OH, MH, and PH salinity zones of the main-stem bay were defined by latitudinal boundaries described previously^9^ and complemented by nine stations in tidal waters of major tributaries (Fig. 1).

*Chl-a* was determined on acetone extracts (80–90%) of particulate material collected by vacuum filtration onto glass-fiber filters (Whatman GF/F or equivalent) with 0.3–0.8 µm nominal pore sizes. Spectrophotometric determinations of *chl-a* using trichromatic equations were made on a Beckman DK-2 or equivalent, and fluorometric *chl-a* measurements were made on a Turner model 110, 111, or Turner Designs model 10 and calibrated by spectrophotometry^9^. Secchi depth was determined as the depth where a 30-cm white disk became invisible when lowered over the side of the research vessel. NO_2_ + NO_3_ was measured using analytical methods for water quality documented by CBP^54–56^ following protocols given by D’Elia *et al*.^57^.

### Statistical analyses

Statistical analyses were conducted using “R” version “Another Canoe” v. 3.3.3. Simple, linear fits for time-series data of log_10_ chl-a, Secchi depth, and NO_2_ + NO_3_ were obtained with the statistical module of Kaleidagraph v. 4.5.2 (Synergy Software, Inc.). Non-linear fits for time-series data were developed using generalized additive models (GAM) in the “R” package ‘mgcv’ and generalized additive mixed models (GAMM) in the package ‘gamm’^58–60^. The “R” package ‘mgcv’ contains GAM functions similar to those designed by T. Hastie in S-Plus, based on a penalized regression-spline approach that includes automatic smoothness selection.

We selected GAM based on previous analyses of water-quality properties for the bay^17,18^, recent comparisons of GAM and weighted regressions of time, discharge and season (WRTDS)^61^ by Beck and Murphy^62^, and flexibility of GAM to add predictor variables relevant to the properties analyzed here. Lag effects of one to several months were tested by adding AR terms in the “R” package gamm as ‘mgcv’ does not support this approach. Analysis of variance (ANOVA) showed no significant differences among models with and without lags, confirming that inclusion of multiple predictor variables for ‘time’ in GAM successfully modeled response variables log_10_ *chl-a*, Secchi depth, and NO_2_ + NO_3_.

Model fits, residuals, flow-adjusted predictions at monthly increments, adjusted R^2^, generalized cross validation (GCV) score, % deviance explained, and p-values for F-statistics were obtained for each model. Climatic effects on response variables were quantified by applying GAM to input files of water-quality properties containing log_10_ monthly SRF, log_10_ monthly SUM, and setting salinity at long-term means, 10^th^, or 90^th^ percentiles. Model predictions in low-flow, “dry” conditions were based on flow terms set at 10^th^ percentiles joined by salinity terms at 90^th^ percentiles; mean-flow predictions were based on flow and salinity terms held constant at their mean values; model predictions in high-flow, “wet” conditions were based on flow terms set at 90^th^ percentiles joined by salinity terms at 10^th^ percentiles. Degrees of smoothing (knots = k) were selected by the “R” package ‘mgcv’ to minimize the GCV score, followed by post-hoc adjustments of “k” for individual terms using the function “gam.check”.

Graphical presentations consisted of observed vs model-fitted values of mean, monthly log_10_ *chl-a*, Secchi depth, and NO_2_ + NO_3_ (Fig. 3a–i), time series of flow-adjusted model predictions (Fig. 4a–i), and aggregated model predictions for the complete time series (Fig. 5a–i). Secular trends for specific periods were computed as percent changes of flow-adjusted predictions. Trend analyses based on GAM predictions at an annual scale of data aggregation followed methods presented by Harding *et al*.^17,18^. Graphical predictions in this paper were prepared with Kaleidagraph 4.5.2 (Synergy Software, Inc.).

## Supplementary information

Supplementary Information
