# Supplementary material for: Long-term trends, current status, and transitions of water quality in Chesapeake Bay
Source: Sci Rep. 2019 Apr 30;9:6709. doi: 10.1038/s41598-019-43036-6 (PMC6491606; doi:10.1038/s41598-019-43036-6)

Supplementary Information

Long-term trends, current status, and transitions of water quality in Chesapeake Bay

Lawrence W. Harding, Jr., Michael E. Mallonee, Elgin S. Perry, W. David Miller, Jason E. Adolf, Charles L. Gallegos, Hans W. Paerl

Item 1 - Observed vs model fits of mean, monthly $\log_{10}$ *chl-a* (mg m^{-3}) for nine tributary stations from 1985 to 2015.

Item 2 - Observed vs model fits of mean, monthly Secchi depth (m) for nine tributary stations from 1985 to 2015.

Item 3 - Time series of flow-adjusted model predictions of Secchi depth (m) for nine tributary stations from 1985 to 2015. *Solid black lines* depict model predictions in mean-flow conditions; *dashed blue lines* show predictions in high-flow conditions; *dashed brown lines* show predictions in low-flow conditions.

Item 4 - Observed vs model fits of mean, monthly $NO_2 + NO_3$ (μM) for nine tributary stations from 1985 to 2015.

Item 5 -Time series of flow-adjusted model predictions of $NO_2 + NO_3$ (μM) for nine tributary stations from 1985 to 2015. *Solid black lines* depict model predictions in mean-flow conditions; *dashed blue lines* show predictions in high-flow conditions; *dashed brown lines* show predictions in low-flow conditions.

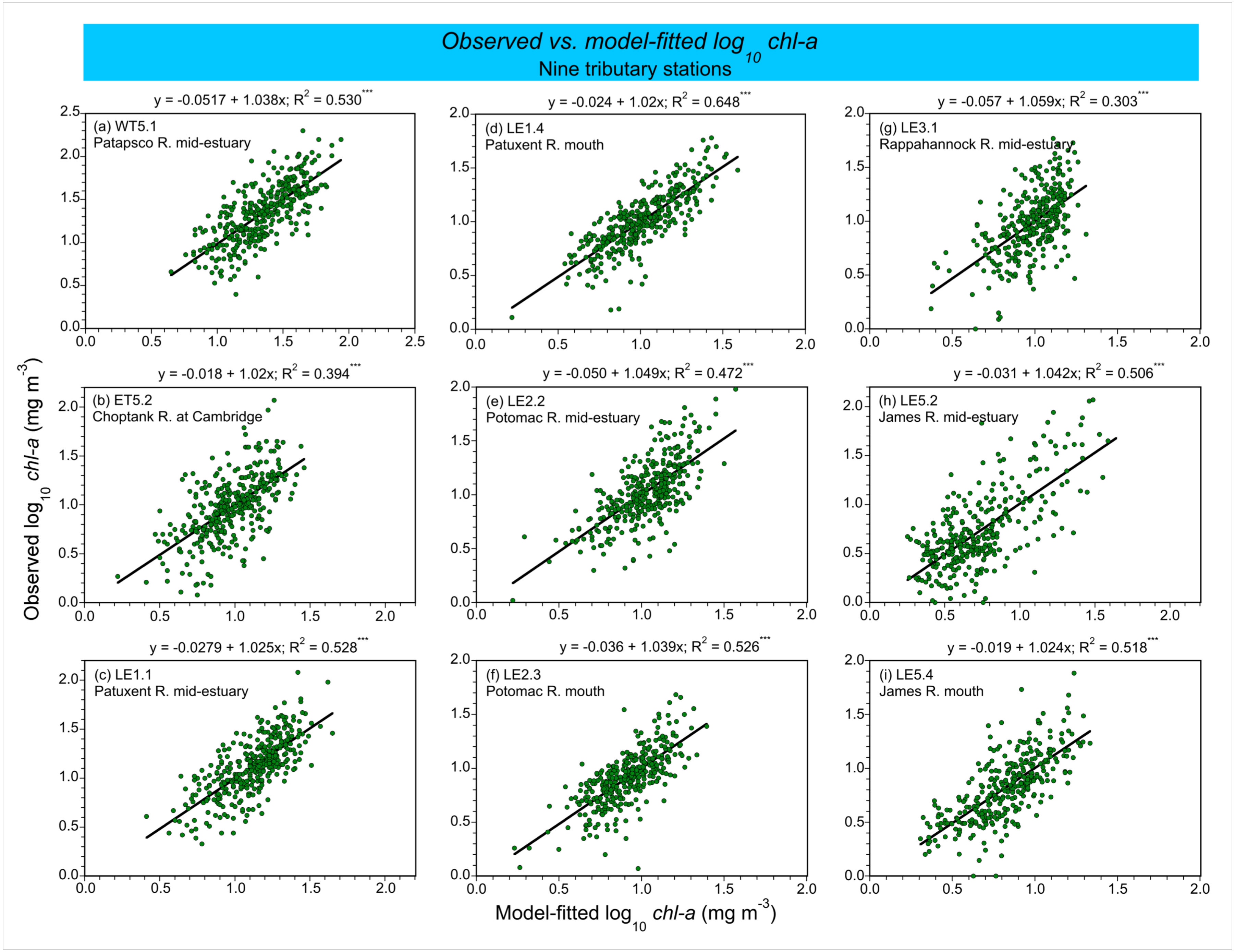

Observed vs. model-fitted log10 chl-a
Nine tributary stations
y = -0.0517 + 1.038x; R2 = 0.530***
(a) WT5.1
Patapsco R. mid-estuary
y = -0.024 + 1.02x; R2 = 0.648***
(d) LE1.4
Patuxent R. mouth
y = -0.057 + 1.059x; R2 = 0.303***
(g) LE3.1
Rappahannock R. mid-estuary
y = -0.018 + 1.02x; R2 = 0.394***
(b) ET5.2
Choptank R. at Cambridge
y = -0.050 + 1.049x; R2 = 0.472***
(e) LE2.2
Potomac R. mid-estuary
y = -0.031 + 1.042x; R2 = 0.506***
(h) LE5.2
James R. mid-estuary
y = -0.0279 + 1.025x; R2 = 0.528***
(c) LE1.1
Patuxent R. mid-estuary
y = -0.036 + 1.039x; R2 = 0.526***
(f) LE2.3
Potomac R. mouth
y = -0.019 + 1.024x; R2 = 0.518***
(i) LE5.4
James R. mouth
Observed log10 chl-a (mg m-3)
Model-fitted log10 chl-a (mg m-3)

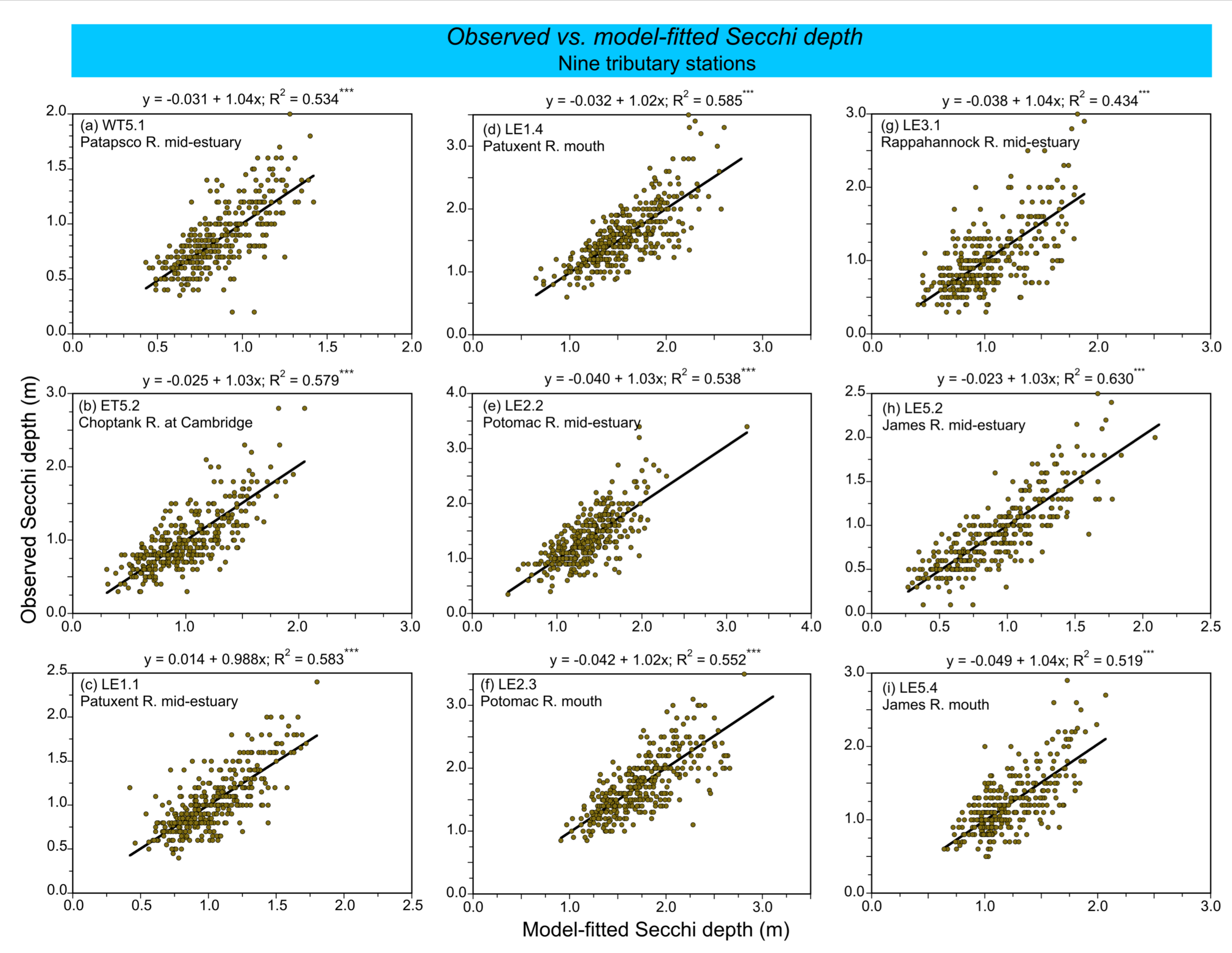
Observed vs. model-fitted Secchi depth
Nine tributary stations
y = -0.031 + 1.04x; R2 = 0.534***
(a) WT5.1
Patapsco R. mid-estuary
y = -0.032 + 1.02x; R2 = 0.585***
(d) LE1.4
Patuxent R. mouth
y = -0.038 + 1.04x; R2 = 0.434***
(g) LE3.1
Rappahannock R. mid-estuary
y = -0.025 + 1.03x; R2 = 0.579***
(b) ET5.2
Choptank R. at Cambridge
y = -0.040 + 1.03x; R2 = 0.538***
(e) LE2.2
Potomac R. mid-estuary
y = -0.023 + 1.03x; R2 = 0.630***
(h) LE5.2
James R. mid-estuary
y = 0.014 + 0.988x; R2 = 0.583***
(c) LE1.1
Patuxent R. mid-estuary
y = -0.042 + 1.02x; R2 = 0.552***
(f) LE2.3
Potomac R. mouth
y = -0.049 + 1.04x; R2 = 0.519***
(i) LE5.4
James R. mouth
Observed Secchi depth (m)
Model-fitted Secchi depth (m)

Time-series of flow-adjusted Secchi depth
Nine tributary stations

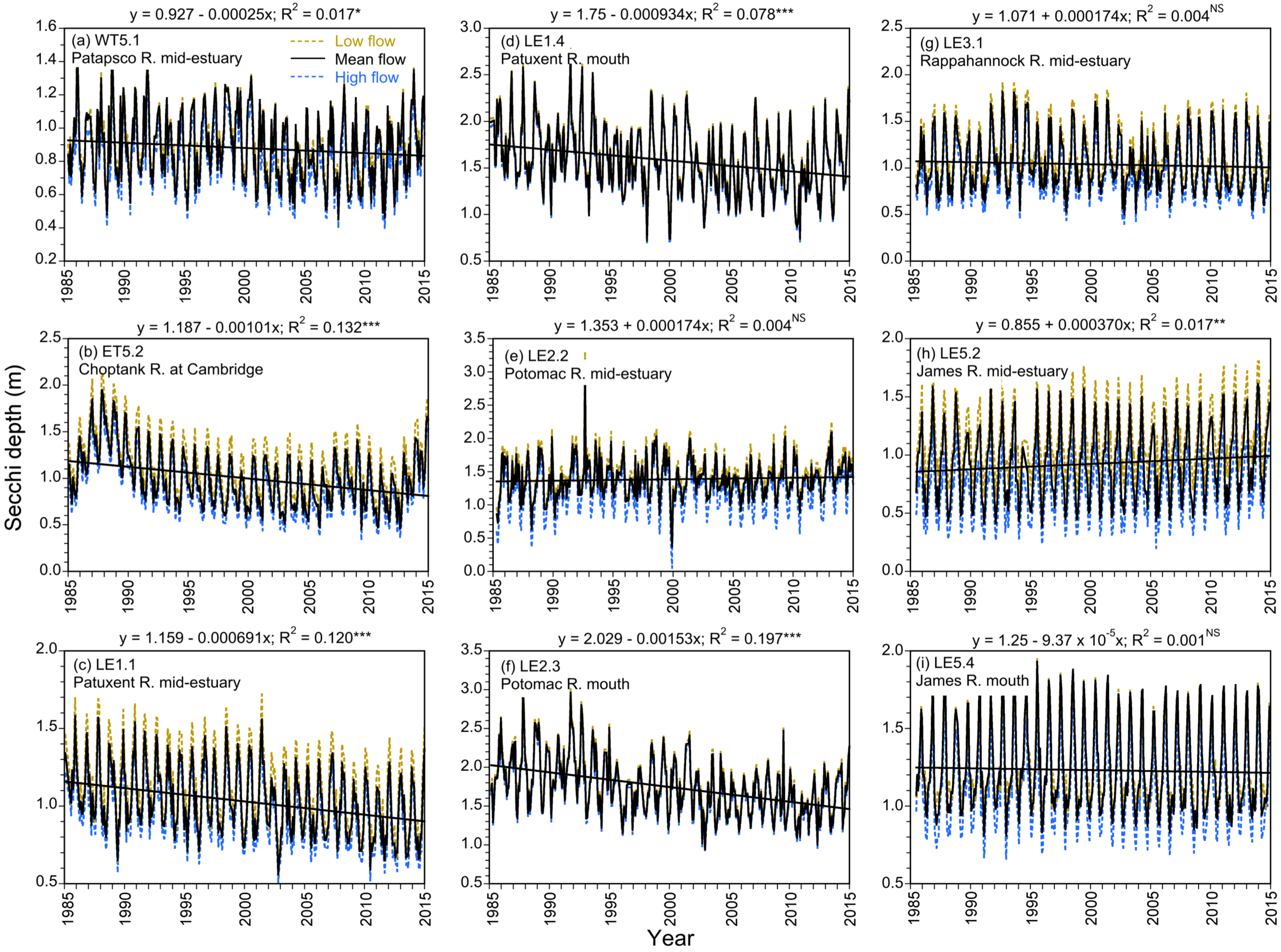

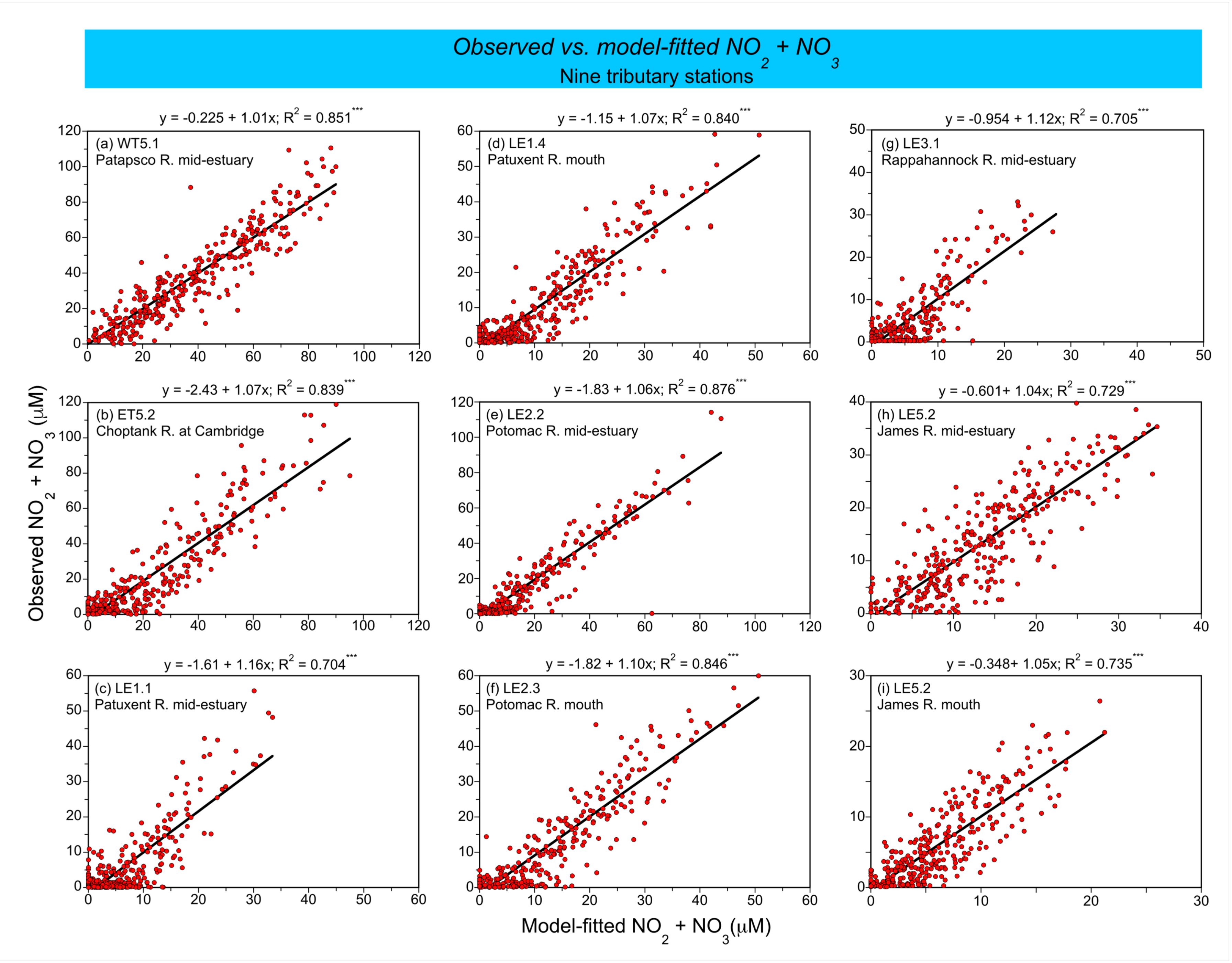

Observed vs. model-fitted NO2 + NO3
Nine tributary stations
y = -0.225 + 1.01x; R2 = 0.851***
(a) WT5.1
Patapsco R. mid-estuary
y = -1.15 + 1.07x; R2 = 0.840***
(d) LE1.4
Patuxent R. mouth
y = -0.954 + 1.12x; R2 = 0.705***
(g) LE3.1
Rappahannock R. mid-estuary
y = -2.43 + 1.07x; R2 = 0.839***
(b) ET5.2
Choptank R. at Cambridge
y = -1.83 + 1.06x; R2 = 0.876***
(e) LE2.2
Potomac R. mid-estuary
y = -0.601+ 1.04x; R2 = 0.729***
(h) LE5.2
James R. mid-estuary
y = -1.61 + 1.16x; R2 = 0.704***
(c) LE1.1
Patuxent R. mid-estuary
y = -1.82 + 1.10x; R2 = 0.846***
(f) LE2.3
Potomac R. mouth
y = -0.348+ 1.05x; R2 = 0.735***
(i) LE5.2
James R. mouth
Observed NO2 + NO3 (μM)
Model-fitted NO2 + NO3 (μM)

Time-series of flow-adjusted $NO_2 + NO_3$ Nine tributary stations

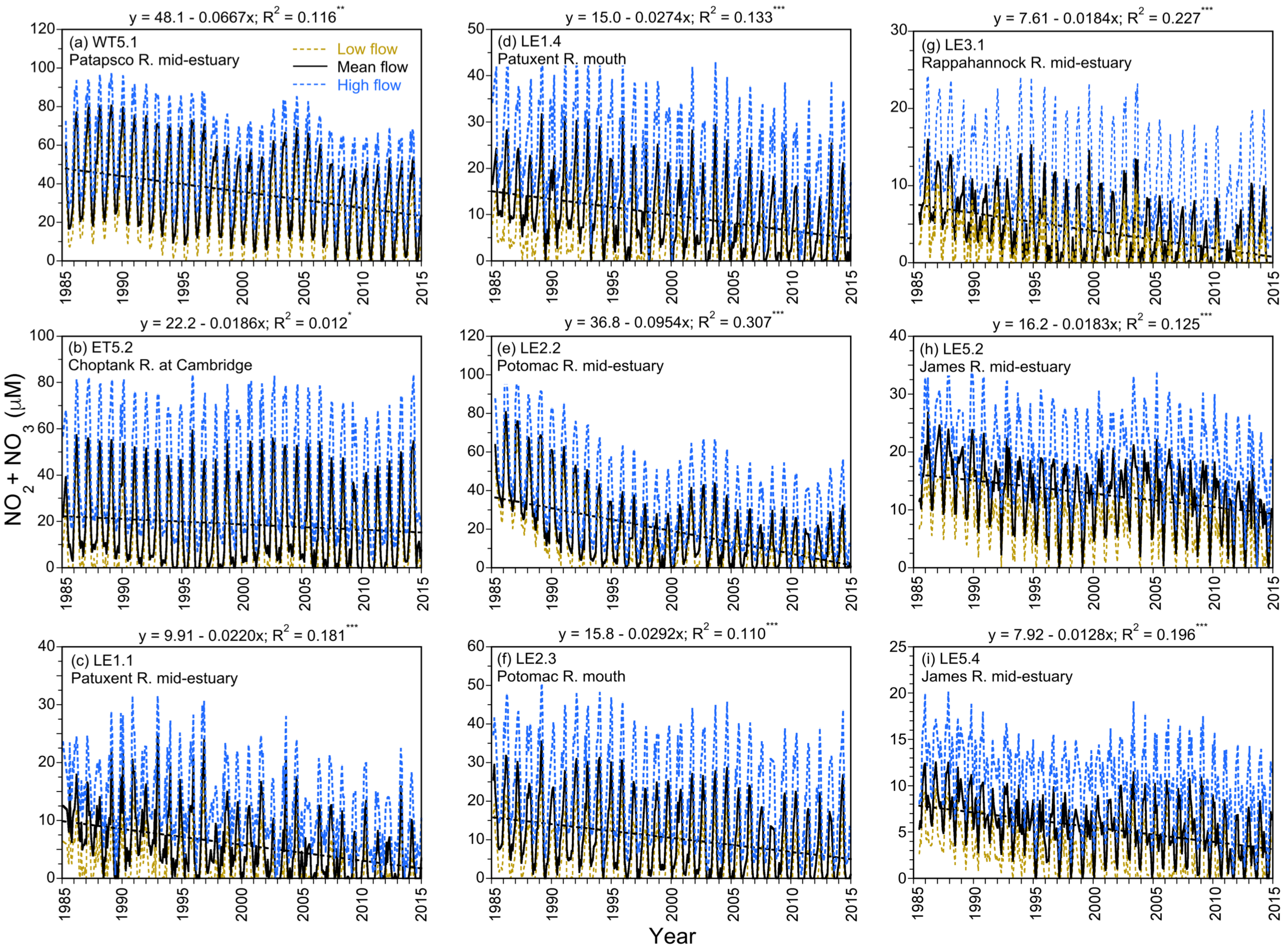

Supplement: Supplementary file 1 — Supplementary Information [file 41598_2019_43036_MOESM1_ESM.pdf]
